# Mathematical Models of Blood-Brain Barrier Transport of Monoclonal Antibodies Targeting the Transferrin Receptor and the Insulin Receptor

**DOI:** 10.3390/ph14060535

**Published:** 2021-06-03

**Authors:** William M. Pardridge, Tom Chou

**Affiliations:** 1 Department of Medicine, UCLA, Los Angeles, CA 90095, USA; 2 Departments of Computational Medicine and Mathematics, UCLA, Los Angeles, CA 90095, USA; tomchou@ucla.edu

**Keywords:** monoclonal antibody, blood-brain barrier, insulin receptor, transferrin receptor, brain drug delivery, biologics

## Abstract

We develop and analyze mathematical models for receptor-mediated transcytosis of monoclonal antibodies (MAb) targeting the transferrin receptor (TfR) or the insulin receptor (IR), which are expressed at the blood-brain barrier (BBB). The mass-action kinetic model for both the TfR and IR antibodies were solved numerically to generate predictions for the concentrations of all species in all compartments considered. Using these models, we estimated the rates of MAb endocytosis into brain capillary endothelium, which forms the BBB in vivo, the rates of MAb exocytosis from the intra-endothelial compartment into brain extracellular space, and the rates of receptor recycling from the endothelial space back to the luminal endothelial plasma membrane. Our analysis highlights the optimal rates of MAb association with the targeted receptor. An important role of the endogenous ligand, transferrin (Tf) or insulin, in receptor-mediated-transport (RMT) of the associated MAb was found and was attributed to the five order magnitude difference between plasma concentrations of Tf (25,000 nM) and insulin (0.3 nM). Our modeling shows that the very high plasma concentration of Tf leads to only 5% of the endothelial TfR expressed on the luminal endothelial membrane.

## 1. Introduction

Biologics are large molecule pharmaceuticals that do not cross the blood-brain barrier (BBB). Therapeutic recombinant proteins, enzymes, decoy receptors, or monoclonal antibodies can be re-engineered as BBB-transportable IgG fusion proteins using molecular Trojan horses [1]. The latter are monoclonal antibodies (MAb) that target endogenous receptor-mediated transport (RMT) systems on the BBB, such as the transferrin receptor (TfR) or insulin receptor (IR). The binding of a TfRMAb or IRMAb to exofacial epitopes on the TfR or IR, respectively, can induce RMT of the antibody across the BBB via transcytosis through the brain capillary endothelium, which forms the BBB in vivo. Such Trojan horse antibodies have entered clinical trials targeting either the human IR [2] or the human TfR1 [3], [NCT04251026, NCT04639050]. A high affinity IRMAb against the human insulin receptor (HIR) was genetically fused to the lysosomal enzyme, iduronidase (IDUA), and this HIRMAb-IDUA fusion protein, designated valanafusp alpha, was tested in a year-long clinical trial in pediatric patients with mucopolysaccaridosis Type I (MPSI), also called Hurler syndrome [2]. A high affinity bivalent TfRMAb, targeting the human TfR1, was genetically fused to another lysosomal enzyme, iduronate 2-sulfatase (IDS), and this TfRMAb-IDS fusion protein, designated pabinafusp alpha, was tested in a phase 2/3 clinical trial in pediatric subjects with MPS Type II (MPSII), also called Hunter syndrome [3]. The IDS enzyme was fused to a monovalent low affinity TfRMAb, also for the treatment of MPSII [4]. Therapeutic antibodies for the brain have also been re-engineered as bispecific antibodies (BSA), where the therapeutic antibody, which alone does not cross the BBB, is genetically fused to a BBB transportable HIRMAb [5] or TfRMAb [6,7,8,9,10,11,12]. BBB penetrating BSAs have also been developed as neurodiagnostics for imaging the brain amyloid [13] or TREM2 [14] in Alzheimer’s disease.

The transcytosis of a receptor-specific MAb Trojan horse through the BBB is a multi-step process that involves binding of the MAb to the targeted receptor on the luminal membrane of the brain capillary endothelium. This complex then undergoes receptor-mediated endocytosis into the intracellular compartment of the endothelium, followed by exocytosis of the MAb across the abluminal membrane of the endothelium that occurs in parallel with receptor recycling from the intra-endothelial compartment back to the luminal membrane of the endothelium. There is little quantitative understanding of the kinetic factors that govern the MAb transport and binding process that includes the effects of e.g., endocytosis, exocytosis, receptor recycling, and MAb-receptor association/dissociation.

The goal of this work is to develop a mathematical model to gain quantitative insight into the kinetics of the multiple steps in the RMT of a Trojan horse MAb across the BBB. By comparing the model predictions to the experimentally observed rates of brain uptake of TfRMAb [15] or IRMAb [16] in primates, we estimated the half-times, *T*_1/2_, of endocytosis, exocytosis, and receptor recycling, as well as the rates of MAb association and dissociation with the TfR or IR. The IR model was tested with HIRMAb-IDUA fusion protein [16]. In consideration of the plasma concentrations of the endogenous ligands for the TfR or IR, it was apparent that separate mathematical models would be required for these two receptors. The TfR on the BBB is >99.99% saturated with the endogenous ligand, transferrin (Tf), owing to the very high concentration of Tf, 45,000 nM, in plasma [17]. In contrast, the plasma insulin concentration, about 0.3 nM in either humans [18] or Rhesus monkeys [19], is nearly 10-fold lower than the equilibrium dissociation constant of insulin binding to the IR, K_D_ = 2.2 nM [20]. Therefore, a TfRMAb is co-endocytosed into the endothelium by a TfR occupied with the endogenous ligand, holo-Tf, whereas the IRMAb is generally endocytosed by the unoccupied IR.

## 2. Results

### 2.1. Transferrin Receptor (TfR) Model

**Figure 1:**
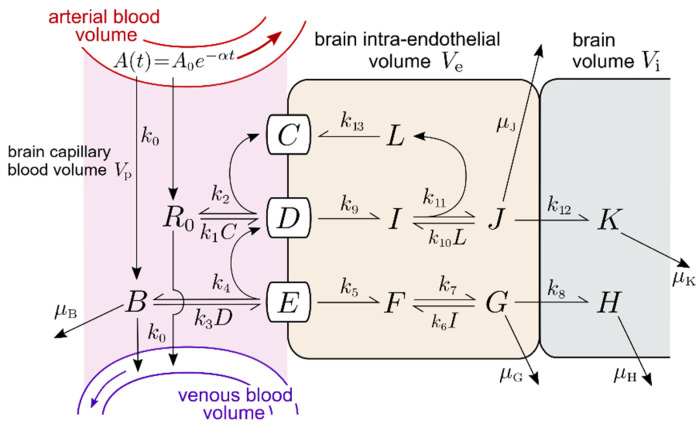
Transferrin receptor (TfR) model. Concentration variables in the relevant tissue compartments are defined in Table 1. Basal parameter values are given in Table 2. *A*(*t*), the MAb concentration in the arterial blood volume after injection reaches maximum value *A*_0_ and is assumed to clear at rate α. The rate *k*_0_ describes the effective transfer rate into and out of the capillary blood volume and depends on the effective flow through the capillary bed. Other association, dissociation, and endocytosis rates are labeled *k*, while degradation/removal rates are labeled μ.

The multicompartment mass-action TfR model outlined in Figure 1 incorporates the rate of cerebral blood flow (CBF) in the brain capillary compartment through the transfer rate *k*_0_. The associated kinetic equations are given in Appendix A, and the corresponding concentration variables are defined in Table 1. Solutions of the concentrations depend on the 23 input parameters and their starting values defined in Table 2. All rate constants have the units of min^−1^ or nM^−1^min^−1^. The units of all output variables are nM and the plasma MAb concentration after injection is given by $A\left(t\right)=A_{0}e^{-at}$. The maximum plasma TfRMAb concentration, *A*_0_, and clearance rate, α, under all injection doses (ID) evaluated in this study have been previously reported in the rhesus monkey [15].

#### 2.1.1. TfR Model Input Parameters

***Brain capillary plasma flow rate constant***. The *k*_0_ parameter depends on brain capillary blood flow and is estimated by *k*_0_ ≈ ln 2/MTT, where MTT is the mean transit time of cerebral blood flow. The MTT is estimated by the *V*_p_/CBF ratio, where *V*_p_ is the brain capillary plasma volume. For the Rhesus monkey, *V*_p_ and CBF are 10 μL/gram [21] and 600 μL/gram/min [22], respectively, which produces an MTT ≈ 1 sec and a *k*_0_ ≈ 42 min^−1^.

***Tf-TfR association and dissociation rate constants***. Binding of iron loaded Tf to the soluble human TfR1 was evaluated by surface plasmon resonance (SPR) [23], which described an association rate *k*_on_ = 1.7 × 10^6^ M^−1^sec^−1^ (equivalent to *k*_on_ = 0.01 nM^−1^min^−1^), and a dissociation rate *k*_off_ = 1.0 × 10^−3^ sec^−1^ (equivalent to *k*_off_ = 0.06 min^−1^). These values were used for the rate constants of holo-Tf association with the TfR, (*k*_1_ and *k*_10_, Figure 1), and the rate constant of Tf dissociation from the TfR (*k*_2_, *k*_11_, Figure 1), as listed in Table 2. The diferric and monoferric forms of holo-Tf in plasma are present in about equal amounts [24], although the affinity of diferric Tf for the TfR1 is about 10-fold greater than the affinity of monoferric Tf [25].

***TfRMAb-TfR association and dissociation rate constants***. The TfRMAb modeled in these studies is a humanized MAb against the human TfR1 [15]. The rate of brain uptake and the plasma pharmacokinetics of this TfRMAb in the adult Rhesus monkey have been previously reported at multiple IDs of 0.2, 3, and 30 mg/kg [15]. This TfRMAb binds to the human TfR1 with an EC50 = 0.36 nM [15], as determined by ELISA performed under conditions where the EC50 approximates the receptor KD [26]. The kinetics of a TfRMAb binding to the TfR-Tf complex have not been reported previously. In this study, binding of the TfRMAb to the Tf-TfR complex, rather than to the unoccupied TfR, is modeled, because the BBB TfR is >99% saturated with endogenous Tf in vivo. Moreover, binding of Tf to the TfR1 induces conformational changes [24], which may affect the binding of a TfRMAb to the receptor. The association rate constant for TfRMAb binding to the TfR-Tf complex at the luminal membrane of the endothelium and in the intra-endothelial compartment are *k*_3_ and *k*_6_, respectively (Figure 1). The rates of dissociation of the TfRMAb from the TfR-Tf complex at the luminal membrane of the endothelium and in the intra-endothelial compartment are *k*_4_ and *k*_7_, respectively (Figure 1). Values of these association and dissociation rates were estimated using numerical simulations of our models. A starting value of *k*_on_ = (*k*_3_, *k*_6_) of 10^6^ M^−1^sec^−1^ (or 0.06 nM^−1^min^−1^) was used as this value was reported for a large panel of MAb’s that bind to a BBB receptor [27]. The starting value of *k*_off_ = (*k*_4_, *k*_7_) was computed from the product *k*_on_·K_D_ = 0.022 min^−1^, where K_D_ = 0.36 nM, and *k*_on_ = 0.06 nM^−1^min^−1^ (Table 2).

***TfR endocytosis rate constant***. The starting rate constant of internalization of the Tf-TfR complex (*k*_9_, Figure 1) or the TfRMAb-Tf-TfR complex (*k*_5_, Figure 1) is 0.14 min^−1^, which is equal to an endocytosis timescale or “half-life” of *T*_1/2_ ≈ 5 min. The timescale of endocytosis of the TfR is *T*_1/2_ ≈ 4–6 min in multiple cell types including human HepG2 cells, Chinese hamster ovary (CHO) cells, and rabbit reticulocytes [28,29,30]. Evidence that internalization of the TfR at the BBB would be comparably fast was reported previously using an internal carotid artery perfusion method coupled with the capillary depletion method and emulsion autoradiography, which demonstrated significant TfRMAb and Tf transcytosis during a 5–10 min internal carotid artery infusion in rats [31].

***TfRMAb and Tf exocytosis rate constant***. The respective starting rate constant, *k*_8_ and *k*_12_, of the exocytosis of the TfRMAb and Tf into the brain extracellular space (ECS) across the endothelial abluminal membrane, is 0.14 min^−1^, which is equivalent to an exocytosis *T*_1/2_ of ~5 min. These values of *k*_8_ and *k*_12_ correspond with prior work showing that both Tf and a TfRMAb rapidly enter the ECS of the brain in vivo following an internal carotid artery infusion of only 5 min (31). Multiple values of *k*_8_, *k*_12_, were tested with the model.

***TfR recycling rate constant***. The starting value of *k*_13_, the rate constant of recycling of the unoccupied TfR from intra-endothelial compartment to the luminal plasma membrane, is 0.14 min^−1^, which corresponds to a TfR recycling *T*_1/2_ ≈ 5 min. The *T*_1/2_ of TfR recycling in HepG2 cells is 17 min [28]. Different values of *k*_13_ were tested with the model.

***Degradation rate constants***. The rate constant, μ_B_, of TfRMAb removal from the brain plasma compartment via mechanisms unrelated to BBB transport, e.g., binding to red blood cells, is set at 0.00096 min^−1^, which corresponds to *T*_1/2_ ≈ 12 h. This value of μ_B_ may be an over-estimate, since the plasma membrane of mature red blood cells does not express the TfR [32]. The rate constants of TfRMAb and Tf degradation in brain, μ_H_ and μ_K_, are also set at 0.00096 min^−1^. The rate constants for Tf and TfRMAb degradation in the endothelium, μ_J_ and μ_G_, were set at 0.0058 min^−1^, which corresponds to a *T*_1/2_ ≈ 2 h. The impact of different values for μ_B_, μ_H_, μ_K_, μ_G_, and μ_J_, was tested in simulations with the model. The model incorporates no rate constants for degradation of the TfR, because it is assumed that the TfR is constant over time due to comparable rates of receptor degradation and synthesis. Chronic treatment of mice with an injection dose (ID) of 3 mg/kg of a high affinity TfRMAb does not alter brain expression of the TfR [33].

***Endothelial TfR concentration***. The concentration of the TfR or IR at the human brain capillary has been determined by quantitative targeted absolute proteomics (QTAP) and is 2.3 ± 0.8 fmol/μg capillary protein and 1.1 ± 0.2 fmol/μg capillary protein, respectively [34]. The concentration of the IR at the cynomolgus monkey brain capillary is 1.5 ± 0.2 fmol/μg capillary protein [35]. The concentration of the TfR1 at the primate brain capillary has not been measured. Since the concentration of the IR at the human and primate brain capillary is comparable, the concentration of the TfR1 at the primate brain capillary is set at 2.3 fmol/μg capillary protein. There is, on average, 162 μg capillary protein per gram brain [36]. Therefore, the amount of capillary TfR1 in the brain is (2.3 fmol/μg capillary protein) × (162 μg capillary protein/gram brain) = 372 fmol/gram brain. The BBB TfR in the brain is expressed in a volume equal to the brain plasma volume (*V*_p_), 10 μL/gram brain [21]. Therefore, the concentration of the TfR at the brain endothelium is (372 fmol/gram brain)/(10 μL/gram brain) = 37 fmol/μL or 37 nM, which is rounded off to 40 nM. For the initial conditions of the model, prior to MAb administration where *A*(*t*) = 0, total TfR at the brain endothelium is distributed in 3 pools: the Tf-TfR complex at the luminal endothelial membrane [variable *D*(*t*)], the Tf-TfR complex within the intra-endothelial compartment [variable *I*(t)], and the unoccupied TfR within the intra-endothelial compartment [variable *L*(t)]. Simulations with *A*(*t*) = 0, and plasma holo-Tf (*R*_0_) = 25,000 nM (Table 2), showed that, in the absence of MAb administration, the steady-state levels of TfR in the *D*, *I*, and *L* pools were 5%, 75%, and 20%, respectively. An identical distribution of the TfR within the *D*, *I*, and *L* pools was observed if R_0_ was reduced 10-fold to 2500 nM, which approximates the holo-Tf in culture medium with 10% serum. Thus, just before MAb is injected, we set the initial conditions at *D*(0) = 2 nM, *I*(0) = 30 nM, and *L*(0) = 8 nM. The concentration of the unoccupied TfR at the endothelial plasma membrane [variable *C*(*t*)] is negligible, owing to the very high concentration of Tf in plasma. The total Tf concentration in plasma is 45,000 nM (17), and about 40% of plasma Tf is apo-Tf [24], which has a very low affinity for the TfR1 at physiologic pH [37]. For these modeling studies, the plasma concentration of holo-Tf available to bind to the BBB TfR1 is set to 25,000 nM (Table 2).

#### 2.1.2. TfR Model Simulations of TfRMAb Uptake by Brain in the Rhesus Monkey

**Figure 2:**
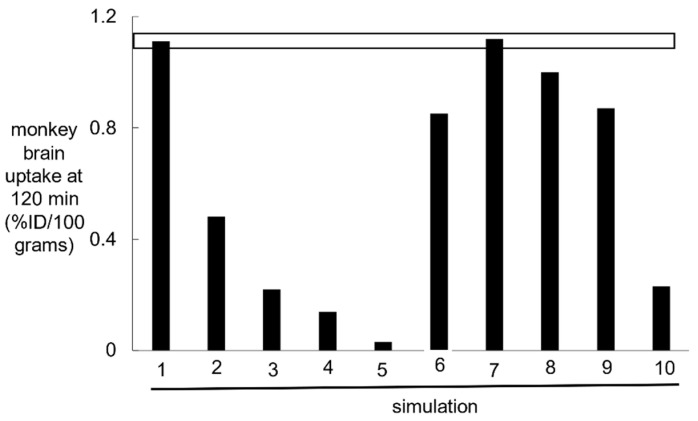
The TfRMAb concentration, expressed as a percent of injected dose (ID) per rhesus monkey brain (100 g), at 120 min following an IV administration of 0.2 mg/kg of the TfRMAb is plotted for simulations 1-10. The 120 min TfRMAb concentrations, in units of nM, are given in Table 3. The brain TfRMAb concentration (nM) is converted to %ID/100 g, based on the ID, 1 mg (6700 pmol), in the 5 kg monkey, the volume of the brain ECS [39], and the weight of the rhesus monkey brain, 100 g [16]. The experimentally determined uptake by the brain of the TfRMAb, at this ID, in the Rhesus monkey has been reported previously [15], and the mean ± SE of this observed uptake is given by the open horizontal bar.

The basal parameters of the TfR model listed in Table 2 were used to predict the concentrations of the 11 model variables at multiple times after IV administration of a high affinity humanized TfRMAb, K_D_ = 0.36 nM, at an ID of 0.15 mg/kg in a 7.4 kg adult Rhesus monkey. This dose totals 1 mg or 6.7 nmol of TfRMAb. Prior work showed the brain uptake of this TfRMAb at this ID, at 120 min after injection, was 1.1 ± 0.1%ID/100 g brain, where the weight of the Rhesus monkey brain is 100 g [15]. Model simulations showed the basal parameters produced TfRMAb concentrations in the intra-endothelial pools of *E* = 1.1 nM, *F* = 20.1 nM and *G* = 0.46 nM at 120 min after IV administration (simulation 1, Table 3). The total TfRMAb concentration in the intra-endothelial compartment at 120 min is *E + F + G*, is 21.7 nM. The units of the intra-endothelial pool were converted to pmol/gram brain from the product of (*E* + *F* + *G*)·*V*_e_, where *V*_e_ is the intra-endothelial volume in brain, which is 0.0008 mL/gram [38]. The TfRMAb concentration in brain [variable *H*(*t*)] is 3.6 nM at 120 min (simulation 1, Table 3). The units of the brain TfRMAb concentration were converted to pmol/gram brain from the product of *H·V*_i_, where *V*_i_, 0.2 mL/gram brain, is the volume of the extracellular space (ECS) in the brain [39]. The total concentration of the TfRMAb in the brain, in units of pmol/gram, is derived from [(*E* + *F* + *G*)·*V*_e_ + *H·V*_i_], and is equal to 0.74 pmol/gram. The total brain TfRMAb concentration, 0.74 pmol/gram is converted to %ID/100g by dividing 0.74 pmol/gram by the ID of 6.7 nmol, which produced a brain uptake of 1.1% ID/100 g. The predicted level of brain uptake, 1.1%ID/100 g, matched the experimentally observed brain uptake shown by the horizontal bar in Figure 2. In simulations 2, 3, and 4, the exocytosis rates *k*_8_ and *k*_12_ were reduced to 0.069 min^−1^ (exocytosis *T*_1/2_ ≈ 10 min), 0.035 min^−1^ (exocytosis *T*_1/2_ ≈ 20 min), and 0.023 min^−1^ (exocytosis *T*_1/2_ ≈ 30 min), respectively, which produced progressive reductions in predicted brain uptake of the TfRMAb relative to the experimentally observed brain uptake. In simulation 5, TfRMAb exocytosis was eliminated from the model with *k*_8_ = 0, and this resulted in a background brain uptake (Figure 2), which reflected only TfRMAb entrapped within the intra-endothelial compartment. In simulation 6, *k*_13_ was reduced to 0.023 min^−1^, which corresponds to a TfR recycling times *T*_1/2_ ≈ 30 min, and the predicted brain uptake of the TfRMAb was reduced relative to the observed value (Figure 2). In simulations 7, 8, and 9, the endocytosis rate, *k*_5_ and *k*_9_, were reduced to 0.069 (*T*_1/2_ ≈ 10 min), 0.035 (*T*_1/2_ ≈ 20 min), and 0.023 (*T*_1/2_ ≈ 30 min), respectively. Comparison of simulations 1 and 7 shows the model predicts an endocytosis *T*_1/2_ of between 5 and 10 min, whereas simulations 8 and 9 show that an endocytosis *T*_1/2_ of 20 or 30 min predicts brain uptake less than what is observed experimentally. In simulation 10, the basal parameters were used, as in simulation 1, except the association rates, *k*_3_ and *k*_6_, of the TfRMAb binding to the Tf-TfR complex, were reduced 10-fold to 0.006 nM^−1^min^−1^ (10^5^ M^−1^sec^−1^). Since the K_D_ was held constant at 0.36 nM, there was a corresponding 10-fold reduction in the dissociation rate constant, *k*_4_ and *k*_7_, of the TfRMAb binding to the Tf-TfR complex to 0.0022 min^−1^ (simulation 10, Table 3). This 10-fold reduction of association and dissociation rates produced an 80% reduction in brain uptake of the TfRMAb (Figure 2).

The simulations described next examine the effect of using TfRMAbs with varying values of K_D_ and association rates *k*_on_.

#### 2.1.3. TfR Model Simulations of the Brain Uptake of a TfRMAb with a High, Moderate, and Low Affinity for the TfR and with Different Association Rate Constants

**Figure 3:**
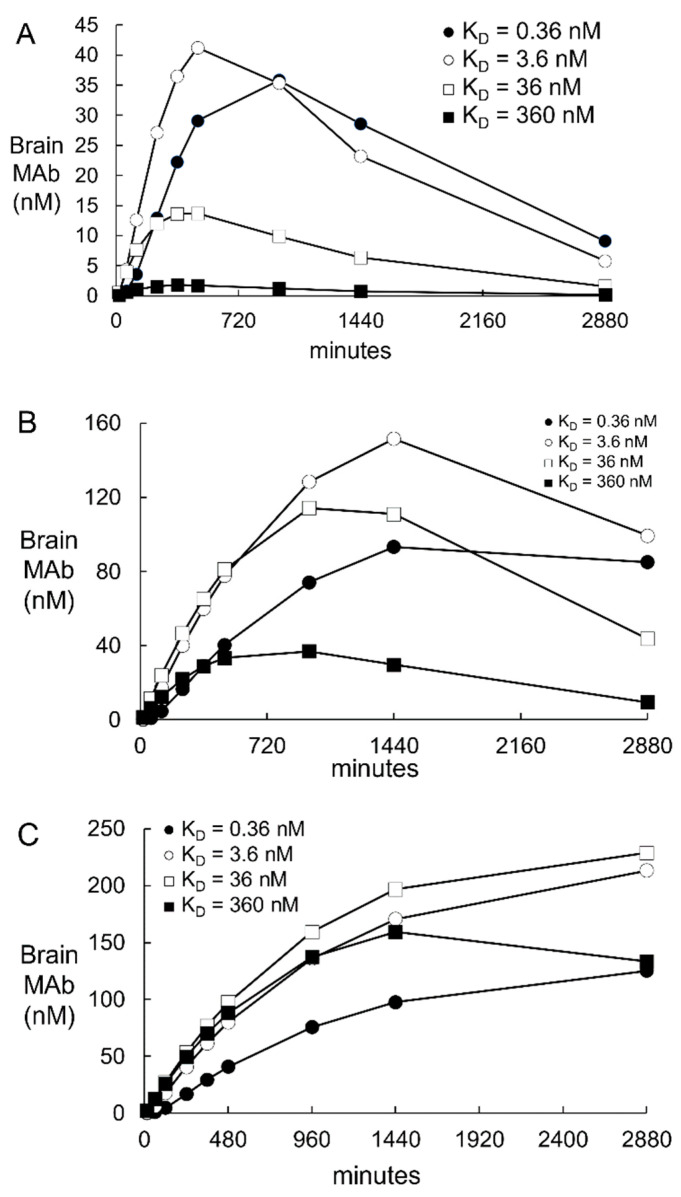
Predicted brain TfRMAb concentrations [variable *H*(*t*), Figure 1] are plotted from *t* = 0 to *t* = 2880 min after an IV administration of 0.2 mg/kg (panel **A**), 3 mg/kg (panel **B**), or 30 mg/kg (panel **C**) of the TfRMAb in the monkey. Simulations were performed for a high affinity TfRMAb (K_D_ = 0.36–3.6 nM), a moderate affinity TfRMAb (K_D_ = 36 nM), and low affinity TfRMAb (K_D_ = 360 nM). The plasma input function, *A*(*t*), was computed from *A*_0_ and α values reported previously for each of these injection doses [15]. The parameter values used for these simulations are those derived from simulation 1 (Figure 2, Table 3).

A high affinity TfRMAb is defined by a K_D_ of 0.36–3.6 nM, a moderate affinity TfRMAb is defined by a K_D_ of 36 nM, and low affinity TfRMAb is defined by a K_D_ of 360 nM. This broad range of affinity of the TfRMAb for the TfR covers the spectrum of TfRMAb BBB Trojan horses that have been developed previously. High affinity TfRMAbs have been described including an 8D3 chimeric TfRMAb with a K_D_ = 2.6 nM for the mouse TfR1 [40], a humanized TfRMAb, with a K_D_ = 0.36 nM for the human TfR1 [15], a human TfRMAb-IDS fusion protein with a K_D_ = 0.12 nM for the human TfR1 and a K_D_ = 0.86 nM for the primate TfR1 [41], and TfRMAb derived from a variable domain of a new antigen receptor, VNAR [42]. Moderate affinity TfRMAbs have been described including a knob-in-hole monovalent BSA with a K_D_ of 37 nM for the primate TfR1 [7], a monovalent BSA with a K_D_ of 34 nM [8], and a dual variable domain bivalent BSA with a K_D_ of 20 nM for the mouse TfR1 [12]. A low affinity TfRMAb was engineered with loss of function and a K_D_ of 111 nM for the mouse TfR1 by mutation of amino acids in the variable region of the antibody [6]. A low affinity monovalent BSA with a K_D_ of 120 nM for the human TfR1 and a K_D_ of 1900 nM for the primate TfR1 was produced by engineering a TfR1 binding site in the near carboxyl terminal region of one heavy chain [11]. Simulations were performed using the TfR model basal parameters (Table 2), which is simulation 1 in Figure 2, with a K_D_ of TfRMAb binding to the TfR1 of 0.36 nM, 3.6 nM, 36 nM, and 360 nM. The *k*_on_ values (*k*_3_, *k*_6_) were fixed at 0.06 nM^−1^min^−1^ and the *k*_off_ values (*k*_4_, *k*_7_) were increased to 0.022, 0.22, 2.2, and 22 min^−1^, in proportion to the K_D_ values 0.36, 3.6, 36, and 360 nM, respectively (Table 4). Simulations were performed for IDs of 0.2, 3, 30, and 50 mg/kg of the TfRMAb. The plasma input function was computed from the *A*_0_ and α values, *A*_0_ = 20 nM, 300 nM, and 3000 nM and α = 0.0055 min^−1^, 0.0021 min^−1^, and 0.0010 min^−1^, respectively, for ID = 0.2 mg/kg, 3 mg/kg, and 30 mg/kg, as reported previously [15]. The *A*_0_ and α values for the 50 mg/kg dose, *A*_0_ = 5000 nM and α = 0.0010 min^−1^, were estimated by extrapolation from parameters reported previously of doses of 3, 10, and 30 mg/kg in the Rhesus monkey [15]. Based on the *A*(*t*) values from *t* = 0 to *t* = 2880 min, the plasma area under the concentration curve (AUC) was computed with the trapezoidal rule and the plasma AUC values for the TfRMAb are given in Table 5 at an ID of 0.2, 3, and 30 mg/kg. The brain TfRMAb concentration [variable *H*(*t*)] from *t* = 0 to *t* = 2880 min is plotted in Figure 3 for the 0.2 mg/kg dose (Figure 3A), the 3 mg/kg dose (Figure 3B), and the 30 mg/kg dose (Figure 3C). The brain concentration curves for each antibody at the 50 mg/kg ID overlapped the brain concentration curves in Figure 3C for the 30 mg/kg ID. The values for the brain AUC over the time period between *t* = 0 and *t* = 2880 min were computed with the trapezoidal method for each TfRMAb, and these brain AUC values are given in Table 4, where the association rates *k*_3_ and *k*_6_ for each antibody are fixed at 0.06 nM^−1^min^−1^. There is no difference in the brain AUC for any antibody between the ID of 30 mg/kg and the ID of 50 mg/kg, except for a 12% increase in brain AUC for the low affinity TfRMAb (K_D_ = 360 nM). At the low ID of 0.2 mg/kg, the highest brain AUC was produced with the high affinity TfRMAbs with a K_D_ = 0.36–3.6 nM. At the middle ID of 3 mg/kg, the brain AUC was highest for the TfRMAb with a K_D_ = 3.6 nM. At the high ID of 30 mg/kg, the brain AUC was highest for the TfRMAb with a K_D_ = 36 nM (Table 4). In a second set of simulations, the association rate constants *k*_3_ and *k*_6_ were fixed for all 4 antibodies at 0.006 nM^−1^min^−1^, and the brain AUC values for each of the 4 antibodies, at each of the 3 IDs, are given in Table 4. There is not a clear relationship between the brain AUC for a given antibody and the dissociation rates *k*_4_ and *k*_7_ which are listed in Table 4 for each antibody. For example, at an ID = 0.2 mg/kg, the brain AUC is 2.6-fold higher for the antibody with a K_D_ of 0.36 nM and a *k*_on_ = 0.06 nM^−1^min^−1^ as compared to the AUC for the antibody with a K_D_ of 3.6 nM and a *k*_on_ = 0.006 nM^−1^min^−1^, although both antibodies have the same dissociation rate *k*_off_ = 0.022 min^−1^. The brain AUC is decreased when the rate *k*_on_ is lowered from 0.06 nM^−1^min^−1^ to 0.006 nM^−1^min^−1^, irrespective of K_D_ or ID (Table 4). The negative impact of a slow *k*_on_ rate on the brain AUC is offset by increasing the ID (Table 4). The rate of TfRMAb binding to the Tf-TfR complex on the luminal endothelial membrane is a function of 3 parameters: the concentration of the Tf-TfR complex at the luminal membrane [variable *D*(*t*)], the on-rate *k*_3_, and the TfRMAb concentration in the capillary plasma [variable *B*(*t*)]. An increase in the ID from 0.2 to 3 or 30 mg/kg causes an increase in variable *B*(*t*), which offsets the reduction in *k*_3_ from 0.06 nM^−1^min^−1^ to 0.006 nM^−1^min^−1^ (Table 4).

#### 2.1.4. TfR Model Simulations of the Time Course of All Model Variables

**Figure 4:**
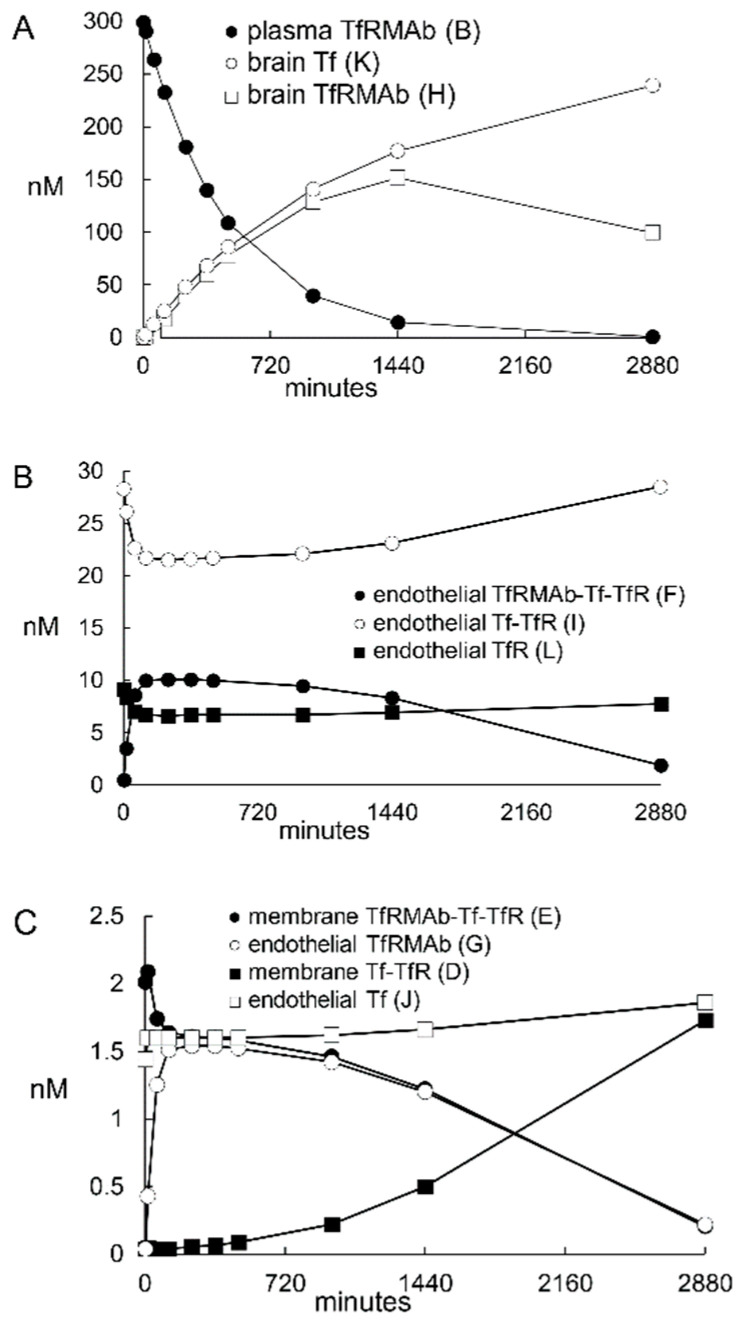
The concentration of the TfR model variables *B*(*t*), *K*(*t*), and *H*(*t*) (**A**), variables *F*(*t*), *I*(*t*), and *L*(*t*) (**B**), and variables *E*(*t*), *G*(*t*), *D*(*t*), and *J*(*t*) (**C**) is plotted from 0 to 2880 min following the IV administration of an ID of 3 mg/kg of a high affinity (K_D_ = 3.6 nM) TfRMAb. The plot shows the concentrations of variables *D*(*t*), *I*(*t*), and *L*(*t*) approach the initial conditions of *D*(0) = 2 nM, *I*(0) = 30 nM, and *L*(0) = 8 nM at 2880 min after TfRMAb administration.

The time course between *t* = 0 and *t* = 2880 min for all model variables is plotted in Figure 4 for the high affinity TfRMAb (K_D_ = 3.6 nM) at an ID = 3 mg/kg. The plasma input function, *A*(*t*), was computed from the previously reported values for *A*_0_ = 300 nM and α = 0.0021 min^−1^, for an ID of 3 mg/kg for the TfRMAb in the primate [15]. This time course shows that by *t* = 2880 min, the values for the *D*, *I*, and *L* variables begin to approach the initial conditions of *D*(0) = 2 nM, *I*(0) = 30 nM, and *L*(0) = 8 nM. Values for variable *C*, the unoccupied TfR at the luminal endothelial membrane (Figure 1) are < 0.0002 nM at all time points, as shown in Table 3. The brain TfRMAb [variable *H*(*t*)] peaks at 1440 min and then decays with a *T*_1/2_ of 12 h, given a μ_H_ value of 0.00096 min^−1^. This value for μ_H_ correlates with a *T*_1/2_ ≈ 12–18 hrs of a TfRMAb in the primate brain [7]. In other studies, the brain TfRMAb concentration decays with a *T*_1/2_ of about 2 days [12,43,44]. The brain TfRMAb [variable *H*(*t*)] may decline with time owing to either antibody degradation in the brain, or by antibody efflux from brain back to blood via the neonatal Fc receptor (FcRn) [5]. The concentrations of membrane TfRMAb-Tf-TfR complex [variable *E*(*t*)], the intra-endothelial TfRMAb-Tf-TfR complex [variable *F*(*t*)], and the free intra-endothelial TfRMAb [variable *G*(*t*)] peak at 15 min, 360 min, and 360 min, respectively. The concentration of Tf in the brain [variable *K*(*t*)] continues to increase with time (Figure 4A). With the basal value of μ_K_ of 0.00096 min^−1^ (*T*_1/2_ = 12 hrs), the brain Tf increases from 0 at *t* = 0 to 250 nM at *t* = 2880 min, and does not exceed a brain concentration above 280 nM even at *t* = 30 days. The concentration of Tf in the adult brain is nearly 8-fold higher, 2000 nM [45]. Simulations showed that a steady state brain Tf concentration of 1900 nM was achieved by 20 days upon reduction of μ_K_ to 0.00014 min^−1^ (*T*_1/2_ ≈ 82 hrs). This estimate of Tf turnover in the brain correlates with the *T*_1/2_ ≈ 2.5 days of Tf turnover in plasma [46]. Simulations evaluated the effect of an increase in endothelial degradation of the TfRMAb, as reflected in the μ_G_ parameter (Figure 1). Reducing μ_G_ from 0.0058 min^−1^ (endothelial degradation time *T*_1/2_ = 2 hrs) to 0.0024 min^−1^ (endothelial degradation time *T*_1/2_ = 4 hrs) had no effect on the brain TfRMAb concentration. Increasing μ_G_ to 0.023 min^−1^ (*T*_1/2_ ≈ 30 min) reduced the peak brain TfRMAb concentration by <10%, irrespective of antibody K_D_ or ID. Increasing μ_G_ to 0.138 min^−1^ (*T*_1/2_ ≈ 5 min) reduced the peak brain TfRMAb concentration by 38–48% (mean 43%), irrespective of antibody K_D_ or ID.

### 2.2. Insulin Receptor (IR) Model

**Figure 5:**
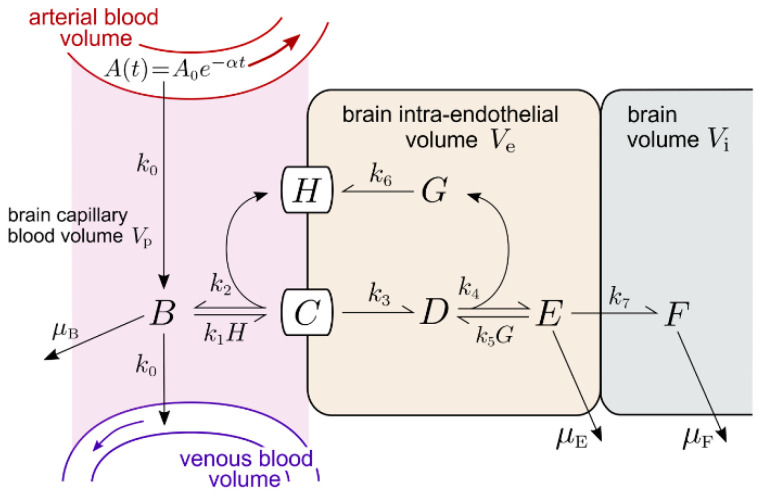
Insulin receptor (IR) model. Variables are defined in Table 6. Basal parameter values are given in Table 7.

The IR model is outlined in Figure 5 and allows for an IRMAb binding to the unoccupied IR on the luminal membrane of the brain capillary endothelium. The IR model is described by the differential equations in Appendix B, and the variables of the IR model are defined in Table 6. The values of the 7 output variables of the IR model are a function of the 14 input parameters, which are defined in Table 7, which also lists the starting value for each of the input parameters. As with the TfR model, all rate constants of the IR model have the units of min^−1^ or nM^−1^min^−1^. The unit of all output variables is nM. The IR model is tested with the HIRMAb-IDUA fusion protein. The plasma input function is defined as described for the Tf model, where values for *A*_0_ and α, at all injection doses (ID) evaluated in this study, have been reported previously for the HIRMAb-IDUA fusion protein following IV administration to the Rhesus monkey [16,47].

#### 2.2.1. IR Model Input Parameters

The basal or starting values for the rate constants of IR endocytosis, IR recycling, and IRMAb exocytosis are identical to these values determined for the TfR model (simulation 1, Figure 2). The endocytosis rate constant, *k*_3_, is set at 0.14 min^−1^; the rate constant for the IR recycling, *k*_6_, is set at 0.035 min^−1^, and the rate constant for the IRMAb exocytosis, *k*_7_, is set at 0.14 min^−1^ (Table 7). The association rate constant for a MAb binding to the HIR is 1.0 × 10^5^ M^−1^sec^−1^ (20), which corresponds to a *k*_1_ and *k*_5_ of 0.006 nM^−1^min^−1^ in the IR model (Figure 5). The K_D_ of binding of the HIRMAb-IDUA fusion protein is 0.93 ± 0.06 nM [48]. The *k*_off_ (*k*_2_, *k*_4_, Figure 5) is derived from the *k*_on_·K_D_ product, and is 0.0056 min^−1^ (Table 7). The starting values for μ_B_, μ_E_, and μ_F_ are also taken from the TfR model (Table 7). The initial simulation examines the brain uptake of the HIRMAb-IDUA fusion protein in the Rhesus monkey following an IV administration of 0.1 mg/kg in a 3.8 kg Rhesus monkey; the A_0_ and α values for this ID have been reported previously [16], and are 2 nM and 0.0173 min^−1^, respectively (Table 7). The concentration of the IR at the cynomolgus monkey brain capillary has been determined by QTAP, and is 1.5 ± 0.2 fmol/μg capillary protein [35]. The amount of brain capillary IR in the brain is (1.5 fmol/μg capillary protein) × (162 μg capillary protein/gram brain) = 243 fmol/gram brain. The BBB IR in the brain is expressed in a volume equal to the brain plasma volume (*V*_p_), 10 μL/gram brain [21]. Therefore, the concentration of the IR at the brain endothelium is (243 fmol/gram brain)/(10 μL/gram brain) = 24 fmol/μL or 24 nM. The concentration of the IR binding sites on the plasma membrane of human brain capillary endothelium, 0.9 fmol/μg capillary protein [49], is about 50% of the total IR. Therefore, for the initial conditions of the model, prior to MAb administration, and *A*(*t*) = 0, total IR at the brain endothelium is distributed in two pools: the unoccupied IR at the luminal endothelial membrane [variable *H*(*t*), Figure 5], and the unoccupied IR within the intra-endothelial compartment [variable *G*(*t*), Figure 5]. For initial conditions, *H*(0) = 12 nM and *G*(0) = 12 nM.

#### 2.2.2. IR Model Simulations of HIRMAb-IDUA Uptake by Brain in the Rhesus Monkey

**Figure 6:**
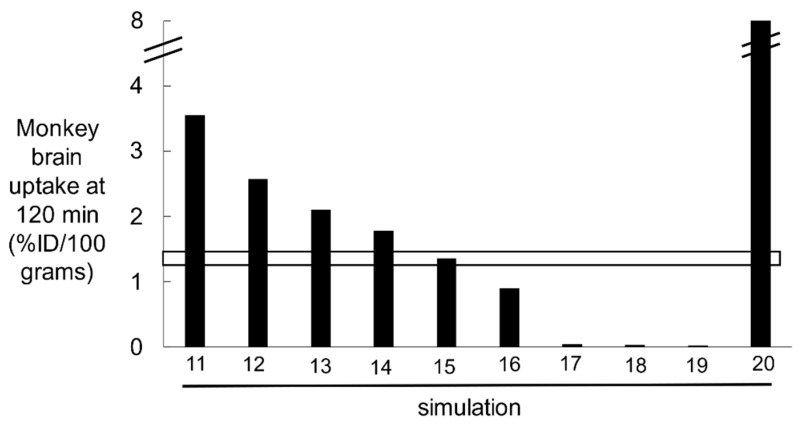
The brain HIRMAb-IDUA fusion protein uptake values, expressed as a percent of injected dose (ID) per rhesus monkey brain (100 g), at 120 min following an IV administration of 0.1 mg/kg of the HIRMAb-IDUA fusion protein are shown for simulations 11–20. The 120 min HIRMAb-IDUA fusion protein concentrations, in units of nM, are given in Table 8. The brain HIRMAb-IDUA fusion protein concentration (nM) is converted to %ID/100 g, based on the ID, 0.38 mg (1.3 nmol), in the 3.8 kg monkey, the volume of the brain ECS (39), and the weight of the rhesus monkey brain, 100 g [16]. The experimentally determined uptake by the brain of the HIRMAb-IDUA fusion protein, at this ID, in the Rhesus monkey has been reported previously [16], and the mean ± SE of this observed uptake is given by the open horizontal bar.

The basal parameters of the IR model listed in Table 7 were used to predict the concentrations of the 7 model variables at 120 min after the IV administration of HIRMAb-IDUA fusion protein, at an ID of 0.1 mg/kg in a 3.8 kg adult Rhesus monkey. This ID is equal to 0.38 mg of the 300,000 Da HIRMAb-IDUA fusion protein, which is equivalent to an ID of 1.3 nmol. The brain uptake of the HIRMAb-IDUA fusion protein at this ID, at 120 min after injection, is 1.2 ± 0.2 %ID/100 g brain [16]. Model simulations showed the basal parameters produced HIRMAb-IDUA concentrations in the intra-endothelial pools of *C*(*t*) = 0.19 nM, *D*(*t*) = 6.1 nM and *E*(*t*) = 0.22 nM at 120 min after IV administration (simulation 1, Table 8). The total HIRMAb-IDUA concentration in the intra-endothelial compartment, *C*(*t*) + *D*(*t*) + *E*(*t*), is 6.5 nM at 120 min. The units of the intra-endothelial pool were converted to pmol/gram brain from the product of (*C* + *D* + *E*)·*V*_e_, where *V*_e_ is the intra-endothelial volume in the brain, which is 0.8 μL/gram [38]. The HIRMAb-IDUA concentration in the brain [variable *F*(*t*), Figure 5] is 2.3 nM at 120 min (simulation 1, Table 8). The units of the brain HIRMAb-IDUA concentration were converted to pmol/gram brain from the product of *F·V*_i_, where *V*_i_, 0.2 mL/gram brain, is the volume of the extracellular space (ECS) in the brain [39]. The total concentration of the HIRMAb-IDUA in the brain, in units of pmol/gram, is derived from [(*C* + *D* + *E*)·*V*_e_ + *F·V*_i_], and is equal to 0.465 pmol/gram. The total brain HIRMAb-IDUA concentration, 0.465 pmol/gram is converted to %ID/100g by dividing 0.465 pmol/gram by the ID of 1.3 nmol, which produced a brain uptake of 3.5% ID/100 g. This predicted brain uptake is nearly 3-fold higher than the experimentally observed brain uptake shown by the horizontal bar in Figure 6. In simulations 12, and 13, the *k*_3_ endocytosis rate constant was reduced to 0.035 min^−1^ (endocytosis *T*_1/2_ = 20 min), and 0.023 min^−1^ (endocytosis *T*_1/2_ = 30 min), respectively, which lowered the predicted brain uptake of the HIRMAb-IDUA fusion protein (Figure 6). In simulations 14 and 15, the *k*_3_ value of 0.023 min^−1^ was used in conjunction with an exocytosis rate constant, *k*_7_, of 0.069 min^−1^ (exocytosis *T*_1/2_ = 10 min) and *k*_7_ = 0.035 min^−1^ (exocytosis *T*_1/2_ = 20 min), respectively. Simulation 15, where *k*_3_ = 0.023 min^−1^ and *k*_7_ = 0.035 min^−1^ predicted a brain uptake of the HIRMAb-IDUA fusion protein that matched the experimentally observed uptake (Figure 6). In simulation 16, the parameters are identical to simulation 15, except parameter *k*_6_ is reduced to 0.023 min^−1^ (recycling timescale *T*_1/2_ ≈ 30 min); this parameter change causes a 34% decrease in brain uptake as compared to simulation 15, where the recycling *T*_1/2_ ≈ 20 min. In simulation 17, exocytosis was eliminated from the model with *k*_7_ = 0, and this resulted in a background brain uptake (Figure 6), which reflected only HIRMAb-IDUA fusion protein entrapped within the intra-endothelial compartment. In simulation 18, endocytosis was eliminated from the model with *k*_3_ = 0, where the fusion protein was only bound, but not endocytosed, at the luminal endothelial plasma membrane, and this also resulted in a background brain uptake (Figure 6). In simulation 19, the *k*_on_ of fusion protein binding to the IR was reduced to 10^4^ M^−1^sec^−1^, so that the association rate constant (*k*_1_, *k*_5_) was reduced to 0.0006 nM^−1^min^−1^, and the dissociation rate constant (*k*_2_, *k*_4_) was also decreased to 0.00056 min^−1^, so that the K_D_ of binding, 0.93 nM, was held constant. The reduced association rate constant caused a reduction in brain uptake of the fusion protein to background levels (Figure 6). The role of the *k*_on_ of MAb binding to the IR was also studied in simulation 20, where the *k*_on_ was increased to 10^6^ M^−1^sec^−1^, corresponding to an increase in association rate constant (*k*_1_, *k*_5_) to 0.06 nM^−1^min^−1^ and a parallel increase in dissociation rate constant (*k*_2_, *k*_4_) to 0.056 min^−1^, while holding K_D_ constant at 0.93 nM. This rapid *k*_on_ rate constant caused a large increase in the predicted brain concentration of the fusion protein to a level nearly 8%ID/100 g (simulation 20, Figure 6), which is 7-fold above the experimentally observed level of brain uptake (horizontal bar, Figure 6). In summary, the parameter values that predict a brain concentration of the HIRMAb-IDUA fusion protein that corresponds to experimentally observed values are *k*_1_ = *k*_5_ = 0.006 nM^−1^min^−1^, *k*_2_ = *k*_4_ = 0.0056 min^−1^, *k*_3_ = 0.023 min^−1^, *k*_6_ = 0.035 min^−1^, and *k*_7_ = 0.035 min^−1^ (simulation 15, Figure 6, Table 8).

Effects of changes in the degradation rate constants (μ_B_, μ_E_, μ_F_, Figure 5) were evaluated for the IR model. The μ_B_ rate constant, which primarily reflects IRMAb binding to the IR on red blood cells, was set at 0.00096 min^−1^ (*T*_1/2_ = 12 hrs), owing to the much lower expression of the IR on red blood cells as compared to the brain capillary endothelium. The IR number on human red blood cells is 2000 receptors/cell [50]. In contrast, the IR is expressed on monkey brain capillaries at a level of 1.5 pmol/mg capillary protein [35]. Assuming 0.3 mg protein per 10^6^ cells, the IR number on brain capillary endothelium is 2.7 × 10^5^ receptors per cell, which is >100-fold higher than the IR number on red blood cells. The μ_F_ parameter reflects degradation of the HIRMAb-IDUA fusion protein in the brain, and was set at 0.00096 min^−1^ (*T*_1/2_ = 12 hrs), as the turnover of the murine form of the HIRMAb in Rhesus monkey brain was characterized by *T*_1/2_ = 16 h [51]. Increasing μ_E_ to 0.023 min^−1^ (endothelial degradation *T*_1/2_ = 30 min) reduced the peak brain HIRMAb-IDUA fusion protein concentration, at 480 min, by 28%. Increasing μ_E_ to 0.138 min^−1^ (endothelial degradation *T*_1/2_ = 5 min) reduced the peak brain HIRMAb-IDUA fusion protein concentration, at 480 min, by 76%. The impact of the μ_E_ parameter on peak brain IRMAb concentration is greater than that of the μ_G_ parameter on the peak TfRMAb concentration, and this is attributed to the slower rate of exocytosis (*T*_1/2_ = 20 min) for the IR system as compared to the rate of exocytosis (*T*_1/2_ = 5 min) for the TfR system.

**Figure 7:**
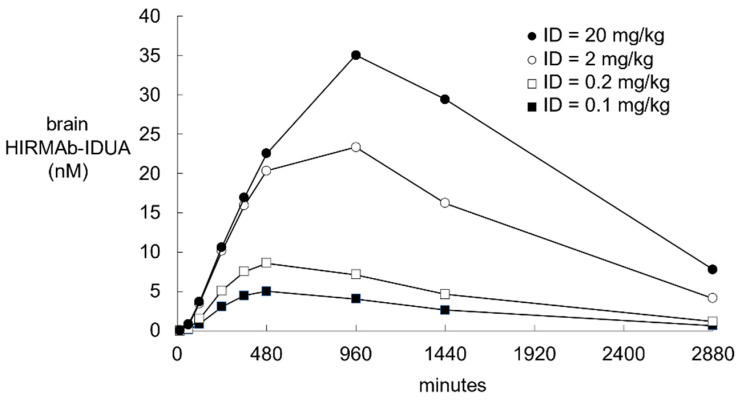
Predicted brain HIRMAb-IDUA fusion protein concentrations [variable *F*(*t*), Figure 5] are plotted from 0 to 2880 min after an IV administration of 0.1, 0.2, 2, or 20 mg/kg of the fusion protein in the monkey. The plasma input function, *A*(*t*), was computed from *A*_0_ and α for each of these 4 injection doses reported previously [16,47]. The parameter values used for these simulations are those derived from simulation 15 (Figure 6, Table 8).

The HIRMAb-IDUA fusion protein has been administered to Rhesus monkeys with an IV injection of 0.1, 0.2, 2, and 20 mg/kg [16,47]. Plasma pharmacokinetics studies showed the *A*_0_ values were 2 nM, 4 nM, 50 nM, and 700 nM, respectively, with corresponding α values of 0.0173 min^−1^, 0.0159 min^−1^, 0.0092 min^−1^, and 0.0084 min^−1^, respectively. These *A*_0_ and α values were used to compute the plasma input function, *A*(*t*). The plasma AUC between *t* = 0 and *t* = 2880 min was computed from the *A*(*t*) values and the trapezoidal rule, and the plasma AUC values are given in Table 5 for an ID of 0.1, 0.2, 2, and 20 mg/kg, respectively. The simulation 15 parameter estimates were used to generate the time course of the brain concentration of the HIRMAb-IDUA fusion protein for each ID (Figure 7). The brain AUC for the HIRMAb-IDUA fusion protein between *t* = 0 and 2880 min was computed with the trapezoidal method, and the brain AUC for the ID of 0.1, 0.2, 2, and 20 mg/kg was 7407 pmol·min/mL, 12,910 pmol·min/mL, 39,273 pmol·min/mL, and 61,050 pmol·min/mL, respectively. The relationship between the ID and the brain AUC is non-linear, consistent with saturation of the brain uptake of the HIRMAb-IDUA fusion protein at high injection doses, owing to the high affinity, K_D_ = 0.93 nM, of the binding of this fusion protein to the insulin receptor.

#### 2.2.3. IR Model Simulations of the Time Course of All Model Variables

**Figure 8:**
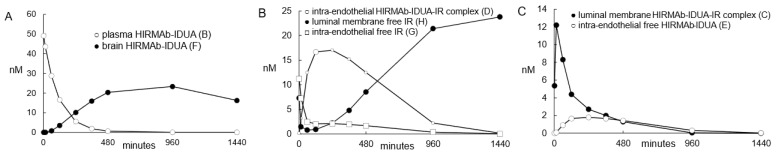
The concentration of IR model variables, *B*(*t*), *F*(*t*) (**A**); *D*(*t*), *H*(*t*), *G*(*t*) (**B**); and *C*(*t*), *E*(*t*) (**C**) is plotted from 0 to 1440 min following the IV administration of an ID of 2 mg/kg of the HIRMAb-IDUA fusion protein. The plot shows the unoccupied IR on the endothelial luminal membrane [variable *H*(*t*), Figure 5] increases to nearly 100% of total endothelial IR at *t* = 1440 min, as the HIRMAb-IDUA fusion protein [variable *B*(*t*), Figure 5] is cleared from plasma.

The time course between *t* = 0 and *t* = 1440 min for all model variables is plotted in Figure 8 for the HIRMAb-IDUA fusion protein at an ID = 2 mg/kg. The plasma input function, *A*(*t*), was computed from the previously reported values for *A*_0_ = 50 nM and α = 0.0092 min^−1^, for an ID of 2 mg/kg of this fusion protein in the primate [47]. This time course shows that by *t* = 1440 min, the concentrations of *B*(*t*), *C*(*t*), *D*(*t*), and *E*(*t*) have all reached negligible values. These findings are consistent with the 22-fold faster rate of plasma clearance of the HIRMAb-IDUA fusion protein in the monkey, 4.1 mL/min/kg at an ID = 2 mg/kg [47], as compared to the plasma clearance of the TfRMAb in the monkey, 0.19 mL/min/kg at an ID = 3 mg/kg [15]. The brain concentration of the fusion protein, variable *F*(*t*), peaks at 960 min and begins to decline, owing to a *T*_1/2_ of 12 hrs for the fusion protein degradation in the brain. The concentration of the unbound IR in the intra-endothelial compartment, variable *G*(*t*), declines toward zero, in parallel with complete recycling of the IR back to the endothelial luminal membrane, variable *H*(*t*) (Figure 8B). The concentration of the unoccupied IR on the endothelial luminal membrane, variable *H*(*t*), approaches the total endothelial IR concentration by *t* = 1440 min (Figure 8B) following the administration of the HIRMAb-IDUA fusion protein. The high concentration of the IR on the luminal endothelial membrane was confirmed by modeling insulin transport across the BBB with the IR model. For this analysis, IR model insulin input parameters for endocytosis, exocytosis, and IR recycling were taken from simulation 15 (Table 8), the *k*_on_ and *k*_off_ parameters of insulin binding to the IR were taken from the literature, 0.006 nM^−1^min^−1^ and 0.013 min^−1^, respectively [20]; other input parameters were *A*_0_ = 0.3 nM (plasma insulin concentration) [18,19], and α = 0. These modeling studies showed the concentration of the IR on the endothelial luminal membrane is 20 nM at steady state in the absence of IRMAb administration. In contrast, as described above, the concentration of the Tf-TfR complex on the endothelial luminal membrane is only 2 nM at steady state in the absence of MAb administration. The 10-fold lower concentration of endothelial luminal Tf-TfR complex, relative to the endothelial luminal IR, is due to the sequestration of the Tf-TfR complex within the intra-endothelial compartment [variable *I*(*t*), Figure 1], which is a consequence of the very large concentration, 25,000 nM, of holo-Tf in plasma (Table 2).

## 3. Discussion

The results of these mathematical model simulations for the BBB receptor-mediated transport (RMT) of a TfRMAb, which targets the BBB TfR, as modeled in Figure 1, and a HIRMAb-IDUA fusion protein, which targets the BBB IR, as modeled in Figure 5, are consistent with the following conclusions. First, it is necessary to develop separate models for the TfR and IR, owing to the very different plasma concentrations of the endogenous ligand, transferrin (Tf) and insulin, respectively. The BBB TfR is > 99% bound by holo-Tf, due to the very high holo-Tf plasma concentration, which is 25,000 nM (Table 2). In contrast, the plasma concentration of insulin, 0.3 nM [18,19], is low relative to the K_D_ of insulin binding to the IR, which is 2.2 nM [20]. Second, fitting previously reported measurements of the brain uptake of a humanized TfRMAb [15], or a HIRMAb-IDUA fusion protein in the Rhesus monkey [16], to the TfR and IR models, respectively, allowed for estimates of the kinetics of endocytosis of the receptor-MAb complex into the intra-endothelial compartment, MAb exocytosis into the brain extracellular space (ECS), and receptor recycling from the intra-endothelial compartment back to the luminal endothelial membrane. Third, simulations fitting known uptake of the MAb in the primate brain to the model allowed for the determination of the optimal rates of MAb association with the receptor on the luminal endothelial membrane. Fourth, the time course of multiple pools in the overall RMT pathways are estimated for the TfR (Figure 4) and the IR (Figure 8), which shows significant differences in the intracellular distributions of the TfR and IR. These differences between the TfR and the IR pathways are linked to the very different plasma concentrations of the endogenous ligands, where the plasma concentration of holo-Tf is >80,000-fold higher than the plasma concentration of insulin. Fifth, based on the estimated rates of MAb association with the luminal receptor, MAb-receptor complex endocytosis, MAb exocytosis, and receptor recycling, the brain TfRMAb exposure was compared at different injection doses (ID), ranging from 0.2–50 mg/kg, for a TfRMAb with high affinity (K_D_ = 0.36–3.6 nM), moderate affinity (K_D_ = 36 nM), and low affinity (K_D_ = 360 nM) for binding to the TfR. Sixth, the plasma AUC is shown to be ~20-fold lower for the HIRMAb-IDUA fusion protein as compared to the TfRMAb (Table 5), which illustrates the impact of the fusion partner, IDUA, on the plasma clearance of the MAb.

The plasma concentration of holo-Tf, 25,000 nM, is >40,000-fold higher than the K_D_, 0.6 nM [23], of Tf binding to the human TfR1, and is 625-fold higher than the total brain endothelial TfR1 concentration, 40 nM (Results and Table 9). In contrast, the plasma insulin concentration, 0.3 nM, is only 1% of the total brain endothelial IR concentration, 24 nM (Results, Table 9). These modeling studies predict there is essentially no free TfR at the endothelial luminal membrane, which forms the BBB in vivo, and that this is a consequence of the very high plasma holo-Tf concentration. In contrast, owing to the low plasma insulin concentration, relative to the total endothelial IR (Table 9), over 90% of the endothelial IR is present on the endothelial luminal membrane (see Results).

Model simulations show the free TfR concentration on the endothelial luminal membrane [variable C(t) in Figure 1] is <0.0002 nM (Table 3), whereas the total TfR at the capillary endothelium is estimated to be 40 nM in the primate (parameter *L*_0_, Table 2). Model simulations with no TfRMAb administration, i.e., *A*_0_ = 0, allowed for modeling of Tf transport through the BBB, and these results showed that at steady state, the concentration of the Tf-TfR complex at the luminal endothelial membrane [variable *D*(*t*), Figure 1], the concentration of the Tf-TfR complex within the intra-endothelial compartment [variable *I*(*t*), Figure 1], and the concentration of the unoccupied TfR that is recycling from the intra-endothelial compartment to the luminal endothelial membrane [variable *L*(*t*), Figure 1] is 2 nM, 30 nM, and 8 nM, respectively, and these results define the initial conditions of the Tf model, ie, *D*(0) = 2 nM, *I*(0) = 30 nM, and *L*(0) = 8 nM (Results). Therefore, the concentration of the Tf-TfR complex at the luminal endothelial membrane, which binds the blood-borne TfRMAb, is only 5% of the total endothelial TfR. This value predicted for the BBB in vivo is lower than the fraction of TfR on the plasma membrane in cultured cells. The plasma membrane fraction of TfR in HeLa cells in tissue culture is 20–30% of the total cellular TfR [52].

Fitting previously reported brain uptake of the TfRMAb in the Rhesus monkey [15] to the model estimated that the rate constants of TfR endocytosis (*k*_5_, *k*_9_, Figure 1), Tf or TfRMAb exocytosis (*k*_8_, *k*_12_, Figure 1), and receptor recycling (*k*_13_, Figure 1) are 0.07–0.14 sec^−1^, 0.14 sec^−1^, and 0.035 sec^−1^, respectively (simulations 1 and 7, Figure 2). These rate constants correspond to a *T*_1/2_ of 5–10 min, 5 min, and 20 min, for TfR endocytosis, TfRMAb exocytosis, and TfR recycling, respectively. These estimates for the TfR are consistent with previously reported rates in cultured cells [28,29,30], as well as previously reported rates of RMT of either Tf or a TfRMAb across the BBB in vivo [31]. The *T*_1/2_ of TfR endocytosis, and TfR recycling, in cultured cells is 4–6 min, and 17 min, respectively [28,29,30]. The *T*_1/2_ of recycling of the asialoglycoprotein receptor in rat liver in vivo is 21 min [53]. Rates of either Tf or TfRMAb exocytosis are known to be fast (on the order of a few minutes), as demonstrated previously with internal carotid artery infusion, coupled with the capillary depletion method and emulsion autoradiography for both Tf and the OX26 TfRMAb in the rat in vivo [31]. Rapid RMT of either Tf or a TfRMAb through the brain capillary endothelium may be attributed, in part, to the very short distance required to complete the transcytosis process in the brain capillary endothelium, which has a cellular thickness of only 0.3 microns [54]. The thickness of the capillary endothelium in the brain is only 3% of the thickness, 10 μm [55], of the choroid plexus epithelium. With respect to the IR, fitting previously reported brain uptake of the HIRMAb-IDUA fusion protein in the Rhesus monkey [16] to the model estimated that the rate constants of IR endocytosis (*k*_3_, Figure 5), exocytosis (*k*_7_, Figure 5), and receptor recycling (*k*_6_, Figure 5) are 0.023 sec^−1^, 0.035 sec^−1^, and 0.035 sec^−1^, respectively (simulation 15, Figure 6). These rate constants correspond to a *T*_1/2_ of 30 min, 20 min, and 20 min, for IR endocytosis, HIRMAb-IDUA exocytosis, and IR recycling, respectively. A *T*_1/2_ of 30 min for IR endocytosis at the BBB corresponds to a *T*_1/2_ of 31 min for endocytosis of the insulin-IR complex in rat liver in vivo [56]. The endocytosis of the HIRMAb by brain capillary endothelium was examined with isolated human brain capillaries. This work showed the *T*_1/2_ of endocytosis was 15–30 min [57], and that the endocytosis of the HIRMAb was an active process in the absence of binding of the endogenous ligand, insulin [57]. The co-administration of the HIRMAb and insulin did not impair the binding of insulin to the IR at the human brain capillary [57]. Transcytosis of HIRMAb fusion proteins across the primate BBB was demonstrated in vivo by emulsion autoradiography of brain removed 120 min following an IV administration of a HIRMAb-lysosomal enzyme fusion protein [58]. Film autoradiography of primate brain at 120 min after IV administration of the HIRMAb-IDUA fusion protein showed global penetration of brain parenchyma by the fusion protein [16]. This high level of brain uptake in the primate would not be possible if there was no transcytosis through the BBB. This was demonstrated in the present modeling studies, which show the level of brain uptake of the fusion protein is background if the high binding and endocytosis at the capillary endothelium is followed by no exocytosis (*k*_7_ = 0, simulation 17, Figure 6). Similarly, if the HIRMAb-IDUA fusion protein is only bound at the luminal membrane IR, but not endocytosed into the endothelium, the level of brain uptake is also background (*k*_3_ = 0, simulation 18, Figure 6).

The rate constant of TfRMAb association with the Tf-TfR complex (*k*_3_, Figure 1) or the rate constant of HIRMAb-IDUA association with the IR (*k*_1_, Figure 5) at the luminal endothelial membrane was demonstrated to have a significant impact on brain delivery. Previously reported brain uptake of the TfRMAb in the primate [15] could only be fit to the model if *k*_3_ = 0.06 nM^−1^min^−1^, which corresponds to a *k*_on_ value of 10^6^ M^−1^sec^−1^ (simulation 1, Figure 2, Table 3). Conversely, if the K_D_ of the high affinity TfRMAb was fixed at 0.36 nM, and the *k*_3_ was reduced 10-fold to 0.006 nM^−1^min^−1^, which corresponds to a *k*_on_ of 10^5^ M^−1^sec^−1^, then the predicted brain uptake of the TfRMAb was reduced 80% (simulation 10, Figure 2, Table 3). High *k*_on_ values that approximate 10^6^ M^−1^sec^−1^ offset the low concentration of the Tf-TfR complex at the luminal endothelial membrane. The lower the *k*_on_ value, the higher the ID needed to produce adequate brain AUC levels (Table 4). If the concentration of the endothelial luminal Tf-TfR complex is 2 nM, the plasma TfRMAb concentration is 264 nM, e.g., 60 min after the administration of 3 mg/kg of the TfRMAb (Figure 4A), the *k*_on_ value is 0.06 nM^−1^min^−1^ and the K_D_ value is 3.6 nM, then the product of (*k*_on_)·(Tf-TfR)·(TfRMAb) is 32 pmol/min/mL. If the *k*_on_ is reduced 10-fold to 0.006 nM^−1^min^−1^, then the product of (*k*_on_)·(Tf-TfR)·(TfRMAb) is reduced 10-fold to 3 pmol/min/mL, but is raised 10-fold to 32 pmol/min/mL if the ID is increased 10-fold and the plasma TfRMAb concentration is raised 10-fold to 2800 nM. A TfRMAb with a reduced *k*_on_ value, irrespective of the K_D_ of TfRMAb binding to the TfR, requires a proportionate increase in ID to produce a therapeutic brain AUC of the antibody (Table 4).

The finding of these modeling studies on the significance of the *k*_on_ value to brain uptake of a TfRMAb illustrates the importance of reliable estimates of association rate constants for TfRMAb binding to the TfR. The *k*_on_ and *k*_off_ values may be estimated by either surface plasmon resonance (SPR) or biolayer interferometry (BLI). BLI enables high throughput measurements but is less sensitive than SPR [59]. The *k*_on_ value for binding of the 8D3 TfRMAb to the mouse TfR1, or the OX26 TfRMAb binding to the rat TfR1, is 5- to 6-fold higher when measured by SPR [42] as compared to BLI [12,60]. The *k*_on_ values are typically determined at 23 °C. However, *k*_on_ and *k*_off_ values are 6- to 7-fold higher at 37 °C, as compared to 23 °C [61,62]. Therefore, *k*_on_ values at physiologic temperature could be under-estimated by nearly 50-fold. Another factor in the extrapolation of *k*_on_ values determined in vitro with SPR to *k*_on_ values in vivo at the brain capillary endothelium is the very different surface density of the immobilized ligand, e.g., TfR, that exists in SPR studies vs the TfR surface density in vivo. The ligand surface density in SPR is typically 1–50 fmol/mm^2^ [63,64]. The TfR surface density of the BBB, 0.03 fmol/mm^2^, can be computed from the concentration of TfR1 at the human or primate brain endothelium, 2 fmol/μg capillary protein [34], the presence of 166 μg capillary protein per gram brain [36], and the capillary surface area in the brain, 120 cm^2^/gram [65]. This estimate of TfR1 density of 0.03 fmol/mm^2^ is an over-estimate of the TfR1 density at the luminal membrane because the latter is only 5% of total endothelial TfR1. Therefore, the TfR1 ligand density in vivo at the brain capillary endothelium is <<3% of the lowest ligand density used in SPR experiments. Since essentially all of the TfR expressed on the endothelial luminal membrane is in the Tf-TfR complex, and not the free TfR (Results), the study of association and dissociation rates of TfRMAb binding to the TfR should include measurements of antibody binding to the complex of holo-Tf and the TfR. Measurement of the kinetics of TfRMAb binding to the Tf-TfR complex is important because Tf binding to the TfR induces conformational changes in the apical domain of the TfR [24,37], which may affect the binding of a TfRMAb to the TfR.

Fitting the experimentally observed level of brain uptake of the TfRMAb (K_D_ = 0.36 nM) in the monkey [15] to the TfR model allowed for estimation of the *k*_on_ value for this antibody in vivo, 0.06 nM^−1^min^−1^ (Figure 3). In contrast to the TfR, the *k*_on_ of the IR ligand, the HIRMAb-IDUA fusion protein, was estimated to be *k*_1_ = 0.006 nM^−1^min^−1^ (simulation 15, Figure 6). If the HIRMAb-IDUA fusion protein *k*_1_ was increased 10-fold to 0.06 nM^−1^min^−1^, then the predicted brain uptake was >7-fold higher than the experimentally observed brain uptake in the primate (simulation 20, Figure 6). These considerations suggest a HIRMAb candidate with a *k*_on_ of 10^5^ M^−1^sec^−1^ is a suitable MAb for targeting the BBB IR, but that a HIRMAb with a *k*_on_ of 10^6^ M^−1^sec^−1^ might provide a higher level of brain uptake (simulation 20, Figure 6). Despite the lower association rate 0.006 nM^−1^min^−1^, for the IR model, the predicted brain uptake of the HIRMAb-IDUA fusion protein matches the experimentally observed brain uptake (simulation 15, Figure 6). In contrast, a 10-fold higher association rate constant, 0.06 nM^−1^min^−1^, for the TfRMAb is required to predict a level of brain uptake that matches the experimentally observed brain uptake (simulations 1 or 7, Figure 2). The higher concentration of luminal membrane IR [variable *H*(*t*), Figure 5], as compared to the concentration of luminal membrane Tf-TfR complex [variable *D*(*t*), Figure 1], offsets the lower association rate constant for binding to the IR.

The types of TfRMAbs that have been developed as BBB Trojan horses cover an affinity spectrum that includes antibodies with high affinity [15,40,41,42], moderate affinity [7,8,10,12], and low affinity [4,6,11] for the TfR. Early TfRMAb Trojan horses were developed with high affinity for either the TfR or the IR [1]. Later, it was hypothesized that low affinity TfRMAbs were preferred owing to a higher brain uptake of a low affinity TfRMAb, K_D_ = 111 nM, as compared to a high affinity TfRMAb, K_D_ = 1.7 nM, following an ID = 20 mg/kg [6]. However, a high affinity TfRMAb was never developed to be administered at such a high ID of 20 mg/kg. This large ID selectively saturates the BBB transport of the high affinity TfRMAb, but has no effect on the saturation of transport of the low affinity TfRMAb. High affinity TfRMAbs were designed to be administered at therapeutic doses of 1–3 mg/kg [1]. Mouse models of neural disease, including lysosomal storage disease, Parkinson’s disease, or Alzheimer’s disease were treated with IV administration of TfRMAb fusion proteins at an ID of 1 mg/kg [1]. A high affinity TfRMAb, at a low ID of 1–3 mg/kg, may produce the same brain exposure of the TfRMAb as a low affinity TfRMAb at a high ID of 20–30 mg/kg. So as to examine the relative brain exposure of a TfRMAb of varying affinity and varying ID, a series of simulations were performed for a TfRMAb with high affinity (K_D_ = 0.36–3.6 nM), moderate affinity (K_D_ = 36 nM), or low affinity (K_D_ = 360 nM) for the TfR1 at an ID of 0.2, 3, 30 mg/kg (Table 4). Simulations were performed for *k*_on_ values of 0.06 and 0.006 nM^−1^min^−1^ (Table 4). The data show that the brain AUC for a high affinity TfRMAb is increased when the ID is increased from 0.2 to 3 mg/kg, but that there is no further increase in brain AUC of a high affinity TfRMAb when the ID exceeds 3 mg/kg (Table 4). Conversely, the brain AUC increases when the ID is increased from 3 to 30 mg/kg for a moderate or low affinity TfRMAb (Table 4), with no further increase in brain AUC caused by increasing the ID from 30 to 50 mg/kg (Results). The affinity of the TfRMAb should be selected based on the intended therapeutic ID. If a target ID of 1–3 mg/kg is planned, then the brain AUC is highest with a high affinity TfRMAb. If the target ID of 30 mg/kg is planned, then the brain AUC is highest with a moderate affinity TfRMAb. The brain AUC is 51% higher following the injection of 30 mg/kg dose of a moderate affinity (K_D_ = 36 nM) TfRMAb as compared to the injection of a 3 mg/kg dose of a high affinity (K_D_ = 3.6 nM) TfRMAb. However, the therapeutic index of the drug may be narrowed when the injection dose is increased 10-fold, and it is not clear that a 50% increase in brain drug concentration is worth the requirement for a 10-fold higher ID. Modeling studies of TfRMAbs of varying K_D_ show that there is no advantage of a low affinity TfRMAb (K_D_ = 360 nM) relative to a moderate affinity TfRMAb (K_D_ = 36 nM) if the intended therapeutic ID is 30 mg/kg (Table 4).

High affinity TfRMAbs have been hypothesized to not undergo exocytosis into brain ECS owing to sequestration within the intra-endothelial compartment due to slow dissociation rates of the TfRMAb from the endothelial TfR [6]. This hypothesis overlooked early in vivo studies showing rapid rates of transcytosis of a high affinity TfRMAb, or Tf, through the BBB in vivo [31]. The absence of TfRMAb exocytosis was examined in the present modeling studies. If there is no exocytosis of the TfRMAb, the brain uptake would be at the background level, and far below the level of brain uptake observed experimentally in the primate (simulation 5, Figure 2). High affinity TfRMAbs are also said to cause down-regulation of the TfR in the brain. However, this is only observed following the IV administration of a very high ID, 50 mg/kg, of a high affinity TfRMAb [66], and this ID is >10-fold higher than the intended ID for a high affinity TfRMAb. When a high affinity TfRMAb is administered to mice either acutely or chronically at an ID of 3 mg/kg, there is no down-regulation of the TfR in the brain, and there is no change in brain iron concentration [33].

The plasma and brain AUC of a TfRMAb that are predicted by the TfR model can be compared with experimentally observed plasma and brain AUC measurements of TfRMAb exposure in vivo. The plasma AUC of the TfRMAb at an ID of 30 mg/kg is predicted by the model to be 2921 nmol·min/mL over 48 hrs (Results), and this AUC is equivalent to 48,683 nmol·hr/L, which extrapolates to 16,227 nmol·hr/L at an ID = 10 mg/kg. This plasma AUC predicted by the model compares well with the plasma AUC of a TfRMAb in the rat, designated OX26-76, which is 20,400 nmol·hr/L at an ID = 10 mg/kg [67]. The brain AUC of the OX26-76, which has a moderate affinity for the TfR (K_D_ = 76 nM), is 1540 nmol·hr/L at an ID = 10 mg/kg in the rat [67]. The brain AUC predicted by the TfR model for the moderate affinity (K_D_ = 36 nM) TfRMAb at an ID of 30 mg/kg is 478 nmol·min/mL (Table 4), which is equivalent to a brain AUC of 7967 nmol·hr/L, which extrapolates to an AUC of 2655 nmol·hr/L at 10 mg/kg. The brain AUC predicted by the model, based on brain uptake in the primate, is 70% higher than the brain AUC observed for the rat [67], which may be due to the lower affinity of the OX26-76 TfRMAb as compared to the moderate affinity (K_D_ = 36 nM) analyzed with the TfR model (Table 4).

The dose–dependent distribution of the HIRMAb-IDUA fusion protein into primate brain is shown in Figure 7. The peak brain concentration of the fusion protein at an ID of 2 mg/kg is 23 nM at 960 min after injection. The molecular weight of the fusion protein, 300 kDa, is twice the MW of the HIRMAb, so that an ID of 2 mg/kg of the fusion protein is equivalent to an ID of the HIRMAb of 1 mg/kg. The peak brain concentration of the TfRMAb (K_D_ = 0.36 nM) at an ID of 3 mg/kg is 93 nM (Figure 3B), which extrapolates to a brain concentration of ~30 nM at an ID = 1 mg/kg. Therefore, the brain delivery of the HIRMAb is comparable to the brain delivery of the TfRMAb, despite the 10-fold lower association rate constant of the HIRMAb-IDUA fusion protein (*k*_1_, *k*_5_, Figure 5), as compared to the association rate constant used in the TfR model (*k*_3_, *k*_6_, Figure 1). There is only a 50% increase in brain AUC of the HIRMAb-IDUA fusion protein when the ID is increased 10-fold from 2 mg/kg to 20 mg/kg (Results). The saturation of the IR delivery system between 2–20 mg/kg is consistent with the high affinity, K_D_ = 0.93 nM, of binding of the HIRMAb-IDUA fusion protein to the IR. There is no advantage to increasing the ID of the HIRMAb-IDUA fusion protein from 2 to 20 mg/kg, because the low ID of 2 mg/kg provides delivery of the IDUA enzyme to brain at a level that normalizes brain IDUA enzyme activity [47]. The plasma AUC for the HIRMAb-IDUA fusion protein is ~22-fold lower than the plasma AUC for the TfRMAb at a comparable ID (Table 5). This accelerated clearance of the HIRMAb-IDUA fusion protein is due to the IDUA domain, since the plasma clearance of a HIRMAb and a TfRMAb in the Rhesus monkey is comparable for an ID of 3–30 mg/kg [15,68]. Fusion of the IDUA enzyme to a mouse-specific TfRMAb causes a 25-fold increase in plasma clearance of the TfRMAb-IDUA fusion protein as compared to the TfRMAb alone in the mouse [40,69]. Fusion of the IDUA enzyme causes a rapid clearance of the fusion protein by the mannose 6-phosphate receptor in peripheral tissues [16]. Since brain AUC is a function of plasma AUC, the role of the fusion partner must be considered in modeling the delivery of biologics to the brain as a TfRMAb or HIRMAb fusion protein.

These mathematical modeling studies predict the kinetics of RMT of a TfRMAb or an IRMAb across the BBB by fitting model predictions to the brain uptake of either a humanized TfRMAb or a HIRMAb-IDUA fusion protein that was determined in vivo for the Rhesus monkey [15,16]. These experimentally observed levels of brain uptake are given by the horizontal bars in Figure 2 and Figure 6 for the humanized TfRMAb and the HIRMAb-IDUA fusion protein, respectively. In both cases, primate brain uptake was measured at 2 h after the IV administration of the TfRMAb or HIRMAb-IDUA fusion protein, which was radiolabeled with [^3^H]-N-succinimidyl propionate or [^125^I]-Bolton-Hunter reagent, respectively. Such radioisotopic methods for determination of brain uptake could over-estimate brain uptake if there was uptake of low molecular weight radiolabeled metabolites, generated by peripheral degradation of the antibody. Yet, prior work showed there was no degradation of the [^3^H]-TfRMAb in plasma [15]. With respect to the [^125^I]-HIRMAb-IDUA, there was a degradation of the fusion protein during the 2 h sampling period [16]. However, low molecular weight [^125^I]-Bolter-Hunter labeled metabolites do not cross the BBB, and do not contribute to brain uptake [70].

## 4. Methods

Separate models for the TfR and IR are outlined in Figure 1 and Figure 5, respectively. The differential equations of the TfR and IR models are given in Appendix A and Appendix B, respectively. The TfR model is comprised of 23 input parameters (Table 2) and 12 output variables (Table 1). The IR model is comprised of 14 input parameters (Table 7) and 8 output variables (Table 6). The models were solved by separate programs using the NDSolve routine of the Wolfram Mathematica program (version 12.2.0.0). The initial conditions of the TfR model are *B*(0) = 0, *C*(0) = 0, *D*(0) = 2 nM, *E*(0) = 0, *F*(0) = 0, *G*(0) = 0, *H*(0) = 0, *I*(0) = 30 nM, *J*(0) = 0, *K*(0) = 0, and *L*(0) = 8 nM. The initial conditions of the IR model are *B*(0) = 0, *C*(0) = 0, *D*(0) = 0, *E*(0) = 0, *F*(0) = 0, *G*(0) = 12 nM, *H*(0) = 12 nM. Variables were estimated at different times after MAb administration, and typically at 15, 60, 120, 240, 360, 480, 960, 1440, and 2880 min. In modeling of Tf transport, brain Tf concentration [*K(t)*, Figure 1] was estimated for up to 30 days (Results). The extent to which the model fit the observed data was made by comparison of the brain TfRMAb concentration [*H(t)*, Figure 1], and the brain HIRMAb-IDUA concentration [*F(t)*, Figure 5], at *t* = 120 min with the experimentally observed values at this time point [15,16]. The experimentally observed level of antibody brain uptake at *t* = 120 min was reported previously for the TfRMAb [15] and the HIRMAb-IDUA [16], and these values are shown by the horizontal bars in Figure 2 and Figure 6 for the TfR model and the IR model, respectively. A series of 10 model simulations were performed for each model and the results of these simulations are given in Table 3 (simulations 1–10) and Table 8 (simulations 11–20) for the TfRMAb and the HIRMAb-IDUA, respectively. For each model, the first simulation, which is simulation 1 for the TfR model (Table 3) and simulation 11 for the IR model (Table 8) is the starting or ‘basal’ simulations for each model, and are based on the parameters given in Table 2 for the TfR model and in Table 7 for the IR model. In simulations 2–10, certain parameters were altered from the basal values of the TfR model, and these altered parameters are given in Table 3. In simulations 11–20, certain parameters were altered from the basal values of the IR model, and these altered parameters are given in Table 8. It is assumed that the TfRMAb or HIRMAb does not interfere with the binding of the endogenous ligand, as demonstrated previously for a TfRMAb [31] and a HIRMAb [57]. Other model assumptions are (i) arterial MAb concentration, *A*(*t*), is equal to the capillary MAb concentration, *B*(t); (ii) there is no receptor degradation or synthesis, as these are offsetting processes, which maintain a constant endothelial receptor concentration in the steady state; (iii) there is minimal constitutive endocytosis of the unbound IR; (iv) the kinetics of MAb binding to the membrane-bound receptor and to the intracellular receptor are identical; (v) the immediate precursor to exocytosis is the free MAb, not the MAb-receptor complex; (vi) MAb efflux from the brain back to blood is incorporated in the brain degradation pathway (μ_H_ for the TfR model or μ_F_ for the IR model). The model does not include pathways of MAb movement through the glial limitans or astrocyte foot processes adhered to the capillary basement membrane. The glial limitans, which covers 63% of the basement membrane in the cryo-fixed brain [71], does not impede protein diffusion. Electron microscopic histochemistry of the brain shows a 40,000 Da peroxidase protein freely traverses the glial limitans to reach the abluminal membrane of the brain capillary endothelium [72].

## 5. Conclusions

Fitting experimentally observed brain uptake of a TfRMAb [15] or a HIRMAb-IDUA fusion protein [16] in the primate to separate models for BBB transport via the TfR and the IR allows for estimates of the kinetics of the individual components (receptor binding, endocytosis, exocytosis, receptor recycling) of RMT of the antibody across the brain capillary endothelium. The typical *T*_1/2_ of these steps range from 5 min to 30 min, and the relatively fast kinetics of the endothelial transcytosis process is consistent with the small thickness, 0.3 microns [54], of the brain endothelium. In the primate brain endothelium, the total TfR and IR are estimated at 40 nM and 24 nM, respectively. The concentration of the TfR endogenous ligand, holo-Tf, is 25,000 nM in plasma [17], which is nearly 1,000-fold higher than the concentration of the endothelial TfR. Conversely, the concentration of the IR endogenous ligand, insulin, 0.3 nM [18,19], is nearly 100-fold lower than the IR concentration at the BBB. Therefore, the major pool of the IR at the endothelial luminal membrane is an unoccupied receptor, whereas all of the TfR at the endothelial luminal membrane is occupied by Tf, and 95% of the endothelial TfR is intracellular, either bound by Tf, or undergoing recycling back to the luminal membrane. Owing to the low concentration of the Tf-TfR complex at the endothelial luminal membrane, a limiting factor in TfRMAb delivery is the association rate constant, *k*_on_, of TfRMAb binding to the Tf-TfR complex, and optimal BBB transport arises when *k*_on_ = 10^6^ M^−1^sec^−1^. In contrast, effective BBB transport of a HIRMAb is observed with a *k*_on_ of 10^5^ M^−1^sec^−1^, and this is attributed to the greater availability of the IR on the endothelial luminal membrane, as compared to the TfR. Finally, the studies show that brain exposure of a high affinity TfRMAb (K_D_ = 0.36 nM) is not increased at an ID above 3 mg/kg. The development of a high affinity, a moderate affinity, or a low affinity TfRMAb as a BBB delivery system is a function of the intended therapeutic dose of the antibody fusion protein. Optimal therapeutic doses of a high affinity TfRMAb are 1–3 mg/kg, whereas the optimal dose of a moderate or low affinity TfRMAb is 10-fold higher, 30 mg/kg. Since Rhesus macaques are genetically closer to humans than other species such as rodents, we analyzed our model using parameters appropriate for macaques. Our models can nonetheless be applied to humans by validating parameters in each individual via measurements employing positron emission tomography and radiolabeled TfRMAb or HIRMAb fusion proteins. Individual-to-individual variability in the model parameters can be studied by imposing distributions over these parameters and/or sensitivity analysis. Our kinetic models can also be extended to the BBB delivery of nanomedicines, such as liposomes or nanoparticles, which are targeted to the brain with receptor-specific antibodies or peptides [73].

## Figures and Tables

**Table 1:** Definitions of concentration variables for TfR model.

*A*(t)	TfRMAb in arterial plasma
*B*(t)	TfRMAb in capillary plasma
*R*_0_	endogenous holo-transferrin (Tf) in arterial/capillary plasma
*C*(t)	unoccupied TfR at brain endothelial luminal membrane
*D*(t)	TfR-Tf complex at endothelial luminal membrane
*E*(t)	TfR-Tf-TfRMAb complex at endothelial luminal membrane
*F*(t)	TfR-Tf-TfRMAb complex in endothelial intracellular compartment
*G*(t)	unbound TfRMAb in endothelial intracellular compartment
*H*(t)	TfRMAb in brain extracellular space
*I*(t)	TfR-Tf complex in endothelial intracellular compartment
*J*(t)	unbound Tf in endothelial intracellular compartment
*K*(t)	Tf in brain extracellular space
*L*(t)	unoccupied TfR in endothelial intracellular compartment

**Table 2:** Parameter definitions and starting values for TfR model.

Parameter	Value	Description
*A*_0_	20 nM	Maximal plasma concentration of TfRMAb after intravenous administration of 0.2 mg/kg
α	0.0055 min^−1^	Rate constant of removal of TfRMAb from plasma by peripheral tissues
*k*_0_	42 min^−1^	Rate constant of brain capillary blood flow
*k*_1_	0.1 nM^−1^ min^−1^	Rate constant of Tf association with TfR at endothelial plasma membrane
*k*_2_	0.06 min^−1^	Rate constant of Tf dissociation from TfR at endothelial plasma membrane
*k*_3_	0.06 nM^−1^ min^−1^	Rate constant of TfRMAb association with Tf-TfR complex at plasma membrane
*k*_4_	0.022 min^−1^	Rate constant of TfRMAb dissociation with Tf-TfR complex at plasma membrane
*k*_5_	0.14 min^−1^	Rate constant of internalization of TfRMAb-Tf-TfR complex from endothelial luminal membrane into intra-endothelial compartment
*k*_6_	0.06 nM^−1^ min^−1^	Rate constant of TfRMAb association with Tf-TfR complex within endothelial cell
*k*_7_	0.022 min^−1^	Rate constant of TfRMAb dissociation with Tf-TfR complex within endothelial cell
*k*_8_	0.14 min^−1^	Rate constant of MAb exocytosis into brain interstitium
*k*_9_	0.14 min^−1^	Rate constant of internalization of Tf-TfR complex from endothelial luminal membrane into intra-endothelial compartment
*k*_10_	0.1 nM^−1^ min^−1^	Rate constant of Tf association with TfR within endothelial cell
*k*_11_	0.06 min^−1^	Rate constant of Tf dissociation from TfR within endothelial cell
*k*_12_	0.14 min^−1^	Rate constant of Tf exocytosis into brain interstitium
*k*_13_	0.035 min^−1^	Rate constant of intracellular TfR recycling back to plasma membrane
μ_B_	0.00096 min^−1^	Rate constant of TfRMAb removal from brain capillary plasma compartment other than binding to endothelial luminal membrane
μ_H_	0.00096 min^−1^	Rate constant of degradation of TfRMAb in brain
μ_K_	0.00096 min^−1^	Rate constant of degradation of Tf in brain
μ_J_	0.0058 min^−1^	Rate constant of degradation of free Tf within endothelial compartment
μ_G_	0.0058 min^−1^	Rate constant of degradation of free TfRMAb within endothelial compartment
*L*_0_	40 nM	Total TfR in endothelial cell
*R*_0_	25,000 nM	Total holo-Tf in plasma

**Table 3:** TfR model simulations of TfRMAb and Tf transcytosis through the BBB over 120 min after intravenous administration in the Rhesus monkey.

Simulation	Parameters	Variable Concentrations (nM)										
		*B*	*C*	*D*	*E*	*F*	*G*	*H*	*I*	*J*	*K*	*L*
1	Basal ^a^	10.3	<0.0002	0.274	1.08	20.1	0.464	3.62	13.3	1.18	22.2	5.29
2	*k*_8_ = *k*_12_ = 0.069 min^−1^	10.3	<0.0002	0.221	0.869	18.7	0.397	1.53	15.9	1.91	15.8	4.27
3	*k*_8_ = *k*_12_ = 0.035 min^−1^	10.3	<0.0002	0.179	0.702	17.2	0.328	0.675	18.4	2.86	10.8	3.46
4	*k*_8_ = *k*_12_ = 0.023 min^−1^	10.3	<0.0002	0.158	0.621	16.5	0.298	0.415	19.6	3.54	8.15	3.03
5	*k*_8_ = 0	10.3	<0.0002	0.252	1.00	23.1	0.762	0	10.7	1.03	21.4	4.84
6	*k*_13_ = 0.023 min^−1^	10.3	<0.0002	0.244	0.958	16.8	0.356	2.76	14.8	1.03	19.4	7.19
7	*k*_5_ = *k*_9_ = 0.069 min^−1^	10.3	<0.0002	0.331	2.34	20.1	0.498	3.65	12.2	1.12	21.5	5.06
8	*k*_5_ = *k*_9_ = 0.035 min^−1^	10.3	<0.0002	0.405	5.040	18.5	0.489	3.26	11.2	1.07	21.1	4.87
9	*k*_5_ = *k*_9_ = 0.023 min^−1^	10.3	<0.0002	0.520	7.69	16.3	0.451	2.82	10.7	1.03	20.8	4.75
10	*k*_3_ = *k*_6_ = 0.006 nM^−1^ min^−1^ *k*_4_ = *k*_7_ = 0.0022 min^−1^	10.3	<0.0002	1.15	0.521	11.0	0.087	0.722	20.6	1.54	25.9	6.61

^a^ Starting parameters for simulation 1 are from Table 2. TfRMAb injection dose ID = 0.2 mg/kg.

**Table 4:** TfRMAb K_D_, binding kinetics, and brain TfRMAb AUC.

ID (mg/kg)	K_D_ (nM) of MAb Binding to TfR	Binding Kinetics and Brain AUC					
		Association *k*_3_, *k*_6_ (nM^−1^min^−1^)	Dissociation *k*_4_, *k*_7_ (min^−1^)	Brain AUC	Association *k*_3_, *k*_6_ (nM^−1^min^−1^)	Dissociation *k*_4_, *k*_7_ (min^−1^)	Brain AUC
0.2	0.36	0.06	0.022	64,524	0.006	0.0022	19,901
	3.6		0.22	64,786		0.022	25,260
	36		2.2	20,127		0.22	14,011
	360		22	2590		2.2	2447
3	0.36	0.06	0.022	204,389	0.006	0.0022	90,081
	3.6		0.22	316,050		0.022	210,015
	36		2.2	233,373		0.22	196,123
	360		22	70,296		2.2	67,412
30	0.36	0.06	0.022	238,284	0.006	0.0022	119,561
	3.6		0.22	420,757		0.022	358,570
	36		2.2	478,087		0.22	457,614
	360		22	358,462		2.2	352,727

Units of brain AUC = (pmol·min/mL).

**Table 5:** Plasma area under the concentration curve (AUC) for TfRMAb and HIRMAb-IDUA fusion protein in Rhesus monkeys.

Injection Dose (mg/kg)	Plasma AUC (pmol·min/mL)	
	TfRMAb	HIRMAb-IDUA
0.1	-	110
0.2	3695	241
2	-	5372
3	148,819	-
20	-	82,813
30	2,921,970	-

Computed from *A*_0_ and α for each injection dose (ID) reported previously for TfRMAb (15) or HIRMAb-IDUA (16, 47).

**Table 6:** Definitions of concentration variables for IR model.

*A*(*t*)	IRMAb in arterial plasma
*B*(*t*)	IRMAb in capillary plasma
*C*(*t*)	IRMAb-IR complex at endothelial luminal membrane
*D*(*t*)	IRMAb-IR complex within endothelial intracellular compartment
*E*(*t*)	unbound IRMAb within endothelial intracellular compartment
*F*(*t*)	IRMAb in brain extracellular space
*G*(*t*)	unoccupied IR within endothelial intracellular compartment
*H*(*t*)	unoccupied IR at brain endothelial luminal membrane

**Table 7:** Parameter definitions and starting values for IR model.

Parameter	Value	Description
*A*_0_	2 nM	Maximal plasma concentration of IRMAb after intravenous administration of 0.1 mg/kg
α	0.0173 min^−1^	Rate constant of removal of IRMAb from plasma via uptake by peripheral tissues
*k*_0_	42 min^−1^	Rate constant of brain capillary blood flow
*k*_1_	0.006 nM^−1^ min^−1^	Rate constant of IRMAb association with IR at endothelial plasma membrane
*k*_2_	0.0056 min^−1^	Rate constant of IRMAb receptor dissociation from IR at endothelial plasma membrane
*k*_3_	0.14 min^−1^	Rate constant of internalization of IRMAb-IR complex from endothelial luminal membrane into intra-endothelial compartment
*k*_4_	0.0056 min^−1^	Rate constant of IRMAb dissociation from IR in intra-endothelial compartment
*k*_5_	0.006 nM^−1^ min^−1^	Rate constant of IRMAb association with IR in intra-endothelial compartment
*k*_6_	0.035 min^−1^	Rate constant of recycling of unoccupied IR from intra-endothelial compartment to luminal plasma membrane
*k*_7_	0.14 min^−1^	Rate constant of free IRMAb exocytosis across endothelial abluminal membrane into brain interstitial volume
μ_B_	0.00096 min^−1^	Rate constant of IRMAb removal from brain capillary plasma compartment other than binding to endothelial luminal membrane
μ_E_	0.0058 min^−1^	Rate constant of degradation of free IRMAb within endothelial compartment
μ_F_	0.00096 min^−1^	Rate constant of removal of free IRMAb from brain via either degradation or efflux back to blood
*L*_0_	24 nM	Total IR in endothelial cell

**Table 8:** IR model simulations of HIRMAb-IDUA fusion protein transcytosis through the BBB over 120 min after intravenous administration in the Rhesus monkey.

Simulation	Parameter Changes from Basal Values	Variables (nM)						
		*B*	*C*	*D*	*E*	*F*	*G*	*H*
11	Basal ^a^	0.251	0.194	6.09	0.224	2.28	16.7	1.04
12	*k*_3_ = 0.035 min^−1^	0.251	1.00	5.27	1.92	1.64	16.8	0.878
13	*k*_3_ = 0.023 min^−1^	0.251	1.62	4.65	0.169	1.34	16.8	0.777
14	*k*_3_ = 0.023 min^−1^ *k*_7_ = 0.069 min^−1^	0.251	1.62	4.69	0.312	1.13	16.9	0.765
15	*k*_3_ = 0.023 min^−1^ *k*_7_ = 0.035 min^−1^	0.251	1.62	4.74	0.508	0.858	16.8	0.75
16	*k*_6_ = 0.012 min^−1^	0.251	1.35	4.15	0.332	0.559	15.0	3.5
17	*k*_7_ = 0 min^−1^	0.251	1.62	4.84	1.16	0	16.8	0.708
18	*k*_3_ = 0 min^−1^	0.251	6.29	0	0	0	17.5	0.180
19	*k*_1_ = *k*_5_ = 0.0006 nM^−1^ min^−1^ *k*_2_ = *k*_4_ = 0.00056 min^−1^	0.251	0.267	0.837	0.0091	0.014	22.7	0.190
20	*k*_1_ = *k*_5_ = 0.06 nM^−1^ min^−1^ *k*_2_ = *k*_4_ = 0.056 min^−1^	0.251	2.72	6.72	2.00	4.94	12.1	2.47

^a^ Starting parameters for simulation 11 are from Table 7. HIRMAb-IDUA injection dose ID = 0.1 mg/kg.

**Table 9:** Comparison of receptor concentration and endogenous plasma concentration for TfR1 and IR.

Parameter	Receptor	
	TfR1	IR
Endogenous ligand	holo-transferrin	insulin
Plasma concentration of endogenous ligand	25,000 nM	0.3 nM
Total endothelial receptor	40 nM	24 nM
[ligand]/[receptor] ratio	625	0.01

## Data Availability

The data presented in this study are available in the Results section.

## Appendix A

The mass-action kinetic equations for the TfR model system depicted in Figure 1 are given by:

(A1a) $$A\left(t\right)=A_{0}e^{-at}$$

(A1b) $$\frac{dB\left(t\right)}{dt}=k_{0}\left(A-B\right)-\left(k_{3}D+\mu_{B}\right)B+k_{4}E$$

(A1c) $$\frac{dC\left(t\right)}{dt}=k_{13}L-k_{1}CR_{0}+k_{2}D$$

(A1d) $$\frac{dD\left(t\right)}{dt}=k_{1}CR_{0}-\left(k_{2}+k_{9}\right)D-k_{3}DB+k_{4}E$$

(A1e) $$\frac{dE\left(t\right)}{dt}=k_{3}DB-\left(k_{4}+k_{5}\right)E$$

(A1f) $$\frac{dF\left(t\right)}{dt}=k_{5}E+k_{6}IG-k_{7}F$$

(A1g) $$\frac{dG\left(t\right)}{dt}=k_{7}F-\left(k_{6}I+k_{8}+\mu_{G}\right)G$$

(A1h) $$\frac{dH\left(t\right)}{dt}=k_{8}G-\mu_{H}H$$

(A1i) $$\frac{dI\left(t\right)}{dt}=k_{9}D+k_{10}LJ-k_{11}I+k_{7}F-k_{6}IG$$

(A1j) $$\frac{dJ\left(t\right)}{dt}=k_{11}I-\left(k_{10}L+k_{12}+\mu_{J}\right)J$$

(A1k) $$\frac{dK\left(t\right)}{dt}=k_{12}J-\mu_{K}K$$

(A1l) $$\frac{dL\left(t\right)}{dt}=k_{11}I-k_{13}L-k_{10}LJ$$

## Appendix B

The mass-action kinetic equations for the IR model system depicted in Figure 5 are given by:

(A2a) $$A\left(t\right)=A_{0}e^{-at}$$

(A2b) $$\frac{dB\left(t\right)}{dt}=k_{0}\left(A-B\right)-\left(k_{1}H+\mu_{B}\right)B+k_{2}C$$

(A2c) $$\frac{dC\left(t\right)}{dt}=k_{1}HB-\left(k_{2}+k_{3}\right)C$$

(A2d) $$\frac{dD\left(t\right)}{dt}=k_{3}C-k_{4}D+k_{5}GE$$

(A2e) $$\frac{dE\left(t\right)}{dt}=k_{4}D-\left(k_{7}+k_{5}G+\mu_{E}\right)E$$

(A2f) $$\frac{dF\left(t\right)}{dt}=k_{7}E-\mu_{F}F$$

(A2g) $$\frac{dG\left(t\right)}{dt}=k_{4}D-k_{6}G-k_{5}GE$$

(A2h) $$\frac{dH\left(t\right)}{dt}=k_{2}C-k_{1}HB+k_{6}G$$

To better understand the parameter dependences in the IR model described by the kinetic equations above, we first note that *C*(*t*) + *D*(*t*) + *G*(*t*) + *H*(*t*) = *L*_0_, the amount total insulin receptor, is conserved. This conservation can be used to eliminate the *H* variable. Furthermore, according to the physiologic parameters given in Table 7, we see that $\alpha /k_{0}\ll 1,\;k_{i}/k_{0}\ll 1$ for *i* = 2,3,4,6,7, and $k_{5}L_{0}/k_{0},\;k_{1}L_{0}/k_{0}\ll 1$. In this realistic limit, we are able to find an analytic approximation that is accurate to within <5% of the exact numerical solution for all times t≫1/α~60 min. This “outer” solution can be expressed in simple terms in which *C*(*t*), *E*(*t*), *F*(*t*), and *G*(*t*) are all proportional to each other:

(A3) $$E\left(t\right)\approx \frac{k_{3}}{k_{7}+\mu_{E}}C\left(t\right),\;F\left(t\right)\approx \frac{k_{3}k_{7}}{\mu_{F}\left(k_{7}+\mu_{F}\right)}C\left(t\right),\;G\left(t\right)\approx \frac{k_{3}}{k_{6}}C\left(t\right),$$

while *D*(*t*) can be approximated by a quadratic polynomial in *C*(*t*):

(A4) $$D\left(t\right)\approx \frac{k_{3}}{k_{4}}C\left(t\right)+\frac{k_{3}^{2}k_{5}}{k_{4}k_{6}\left(k_{7}+\mu_{E}\right)}C^{2}\left(t\right).$$

The membrane-associated concentration of IRMAb-IR, *C*(*t*), is found to be a simple explicit function

(A5) $$C\left(t\right)\approx \frac{v\left(t\right)L_{0}}{2u}\left[\sqrt{1+\frac{4u}{v^{2}\left(t\right)}}-1\right],$$

where the dependences on the parameters arise in the combinations defining *u* and *v*(*t*):

(A6) $$u=\frac{k_{3}^{2}k_{5}L_{0}}{k_{4}k_{6}\left(k_{7}+\mu_{E}\right)},\;v\left(t\right)=1+\frac{k_{3}\left(k_{4}+k_{6}\right)}{k_{4}k_{6}}+\frac{\left(k_{2}+k_{3}\right)e^{\alpha t}}{A_{0}k_{1}}.$$

Thus, the evolution of the concentrations depends only on the combination of parameters given in Equations (A3) and (A6). Specifically, Equations (A3) and (A4) provide simple approximate relationships, among the concentrations evaluated after the initial hour-long transient. Similar asymptotic approximations can be performed for the TfR model (Equation (A1a–j) but results in unwieldy and less informative expressions.
